# Melanoxetin: A Hydroxylated Flavonoid Attenuates Oxidative Stress and Modulates Insulin Resistance and Glycation Pathways in an Animal Model of Type 2 Diabetes *Mellitus*

**DOI:** 10.3390/pharmaceutics16020261

**Published:** 2024-02-09

**Authors:** Sónia Rocha, Andreia Amaro, Marcos D. Ferreira-Junior, Carina Proença, Artur M. S. Silva, Vera M. Costa, Sara Oliveira, Diogo A. Fonseca, Sónia Silva, Maria Luísa Corvo, Marisa Freitas, Paulo Matafome, Eduarda Fernandes

**Affiliations:** 1 Associated Laboratory for Green Chemistry (LAQV), Network of Chemistry and Technology (REQUIMTE), Laboratory of Applied Chemistry, Department of Chemical Sciences, Faculty of Pharmacy, University of Porto, 4050-313 Porto, Portugal; up201607090@edu.ff.up.pt (S.R.); cproenca@ff.up.pt (C.P.); marisafreitas@ff.up.pt (M.F.); 2 Coimbra Institute for Clinical and Biomedical Research (iCBR), Faculty of Medicine, University of Coimbra, 3000-548 Coimbra, Portugal; andreia.amaro15@hotmail.com (A.A.); saraoliveira116@gmail.com (S.O.); diogo.fonseca@ff.uc.pt (D.A.F.); sonias@ci.uc.pt (S.S.); 3 Center for Innovative Biomedicine and Biotechnology (CIBB), University of Coimbra, 3000-548 Coimbra, Portugal; 4 Clinical Academic Center of Coimbra (CACC), 3000-061 Coimbra, Portugal; 5 Department of Physiological Sciences, Institute of Biological Sciences, University Federal of Goiás, Goiânia 74690-900, Brazil; 6 LAQV, REQUIMTE, Department of Chemistry, University of Aveiro, 3810-193 Aveiro, Portugal; artur.silva@ua.pt; 7 Research Unit on Applied Molecular Biosciences (UCIBIO), Laboratory of Toxicology, Department of Biological Sciences, Faculty of Pharmacy, University of Porto, 4050-313 Porto, Portugal; veramcosta@ff.up.pt; 8 Faculty of Pharmacy, University of Coimbra, 3000-548 Coimbra, Portugal; 9 Research Institute for Medicines (iMed.ULisboa), Faculdade de Farmácia, Universidade de Lisboa, 1649-003 Lisbon, Portugal; lcorvo@ff.ulisboa.pt; 10 Coimbra Health School (ESTeSC), Polytechnic University of Coimbra, 3046-854 Coimbra, Portugal

**Keywords:** flavonoid, type 2 diabetes, oxidative stress, advanced glycation end products, prostaglandin E_2_, protein tyrosine phosphatase 1B

## Abstract

Type 2 diabetes *mellitus* (DM) continues to escalate, necessitating innovative therapeutic approaches that target distinct pathways and address DM complications. Flavonoids have been shown to possess several pharmacological activities that are important for DM. This study aimed to evaluate the in vivo effects of the flavonoid melanoxetin using Goto-Kakizaki rats. Over a period of 14 days, melanoxetin was administered subcutaneously to investigate its antioxidant, anti-inflammatory, and antidiabetic properties. The results show that melanoxetin reduced insulin resistance in adipose tissue by targeting protein tyrosine phosphatase 1B. Additionally, melanoxetin counteracted oxidative stress by reducing nitrotyrosine levels and modulating superoxide dismutase 1 and hemeoxygenase in adipose tissue and decreasing methylglyoxal-derived hydroimidazolone (MG-H1), a key advanced glycation end product (AGE) implicated in DM-related complications. Moreover, the glyoxalase 1 expression decreased in both the liver and the heart, correlating with reduced AGE levels, particularly MG-H1 in the heart. Melanoxetin also demonstrated anti-inflammatory effects by reducing serum prostaglandin E_2_ levels, and increasing the antioxidant status of the aorta wall through enhanced acetylcholine-dependent relaxation in the presence of ascorbic acid. These findings provide valuable insights into melanoxetin’s therapeutic potential in targeting multiple pathways involved in type 2 DM, particularly in mitigating oxidative stress and glycation.

## 1. Introduction

Diabetes *mellitus* (DM) has emerged as a public health concern of alarming proportions [1], with approximately 90% of all cases being attributed to type 2 DM [2]. There has been a notable increase in the prevalence of type 2 DM among younger adults, as well as in adolescents and children. This rise has been attributed to the growing rates in obesity [3], as over 80% of individuals with type 2 DM are obese [4]. Type 2 DM is characterized by a reduction in insulin secretion by pancreatic β cells, and by insulin resistance in target tissues, including the liver, muscle, and adipose tissue, ultimately leading to hyperglycemia [5]. Chronic hyperglycemia initiates the activation of multiple signaling pathways across various tissues, leading to a heightened production of reactive pro-oxidant species and oxidative stress, the formation of advanced glycation end products (AGEs), increased cytokine production, and cell death. These processes culminate in the development of various complications associated with DM [6]. At the core of this complex interplay lies oxidative stress, an essential factor intricately involved in the progression of DM and heightened risk of associated complications, particularly in type 2 DM [7,8]. DM complications involve both macrovascular and microvascular issues, including cardiovascular disease (CVD), retinopathy, neuropathy, and chronic kidney diseases. Currently, other complications are being associated with DM, such as nonalcoholic fatty liver disease (NAFLD), depression, hospitalization, and mortality from various infections, such as pneumonia, foot and kidney infections, and cancer, mainly gastrointestinal cancers [3,9]. Specifically, CVD significantly contributes to the elevated risk of premature mortality in individuals diagnosed with type 2 DM. This category of diseases encompasses conditions such as ischemic heart disease, heart failure, stroke, coronary artery disease, and peripheral artery disease, collectively contributing to a mortality rate of at least 50% in individuals with type 2 DM. Hence, these complications pose significant concerns regarding the prognosis of DM [10,11].

A wide range of pharmacological therapies has been approved for the management of DM. However, the approved drugs are associated with several adverse effects, including weight gain, hypoglycemia, gastrointestinal damage, urinary tract infections, and an increased risk of CVD, including heart failure [12]. The alarming prevalence of type 2 DM and future predictive incidence has created a need for novel strategies to effectively manage hyperglycemia, maintain glucose levels within the normal range, and mainly to prevent the onset of DM complications. Nowadays, researchers are challenging the glucose-centric paradigm and focusing on new targets and therapies with additional advantages, such as weight loss capacity, no risk for hypoglycemia, and reductions in cardiovascular and chronic kidney diseases [13,14]. Approaches targeting inflammation and oxidative stress are also novel potential strategies for DM management and its related complications [15,16].

**Figure 1:**
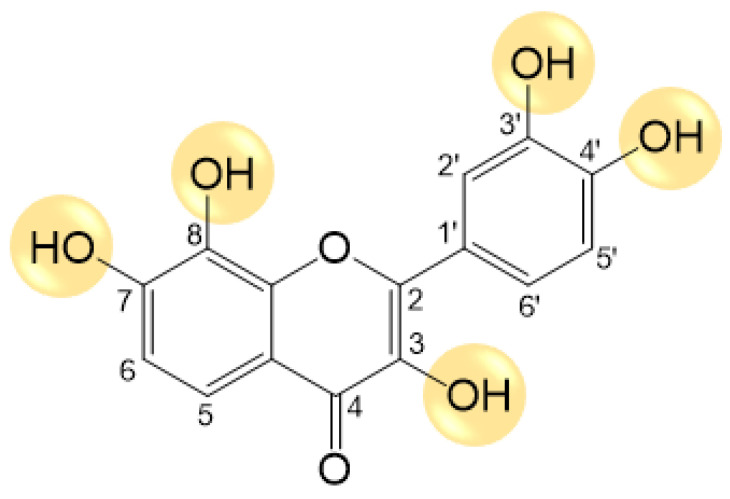
Chemical structure of the studied flavonoid melanoxetin, 3,3′,4′,7,8-pentahydroxyflavone.

Polyphenols, particularly flavonoids, have been recognized for their potential pharmacological activities, particularly as antioxidant [17], anti-inflammatory [18], and antidiabetic agents [19]. Evidence gathered from epidemiological data shows a strong positive correlation between the inclusion of dietary polyphenols and the management/prevention of the onset of type 2 DM [20]. However, until now, no antidiabetic drugs based on the flavonoid skeleton have been approved for clinical use, encouraging further research on this potential scaffold as a new approach to type 2 DM management. The flavonoid melanoxetin, 3,3′,4′,7,8-pentahydroxyflavone (Figure 1), is a naturally occurring flavonoid identified in *Acacia confuse* Merr. (Leguminosae), present in both the wood and the root constituents of the plant. This plant is one of the most widespread species and has been utilized in traditional medicine. Owing to its elevated levels of tannins and phenolic compounds, numerous studies have been conducted in recent years to investigate the phytochemistry of *Acacia confusa* extract [21,22]. Melanoxetin is being studied as a potential xanthine oxidase inhibitor in in vitro experiments and in vivo models using hyperuricemic mice [23,24]. Melanoxetin also demonstrated potential as an anti-inflammatory compound by reducing prostaglandin E2 (PGE_2_) production, and by suppressing the gene expression of inducible ^●^NO synthase (iNOS) and cyclooxygenase-2 (COX-2) [25]. This flavonoid also showed potential in vitro antidiabetic activity as an α-glucosidase inhibitor [26,27]. Furthermore, melanoxetin demonstrated the capacity to reduce reactive oxygen species (ROS) production in a cellular model [28]. Despite its potential, the specific effects of melanoxetin as an antidiabetic, anti-inflammatory, and antioxidant agent in vivo remain relatively unexplored. To the best of our knowledge, the in vivo effects of melanoxetin in models of DM have not yet been explored. Further research is thus required to fully understand its therapeutic potential in DM management, as well as in inflammation and oxidative stress related to DM. Therefore, in the present study, the flavonoid melanoxetin was administered subcutaneously over 14 days in Goto-Kakizaki (GK) rats, an animal model of type 2 DM, to explore its antioxidant, anti-inflammatory, and antidiabetic effects.

## 2. Materials and Methods

### 2.1. Synthesis of Melanoxetin

Melanoxetin was synthesized as previously described [29]. Briefly, the flavonoid was synthesized through a four-step methodology, beginning with the protection of the hydroxyl groups in the starting materials. A solution of 1 M BBr_3_ in CH_2_Cl_2_ (2.0 mL, 2.0 mmol, 2.5 equiv for each protecting group to be cleaved) was gradually introduced into a cooled solution (IPA cryostat bath at —78 °C) of polyalkoxyflavon-3-ol (102 mg, 0.20 mmol) in freshly distilled CH_2_Cl_2_ (7.5 mL). The reaction mixture was stirred under N_2_ at room temperature for 48 h. Subsequently, it was slowly added to ice (5 g) and H_2_O (15 mL) and thereafter vigorously stirred until precipitation was noted. The resulting solid was removed via filtration, extensively washed with H_2_O, and dried in an oven at 55 °C.

Yellow solid (33 mg, 55% yield). Mp 304–307 °C. ^1^H NMR (500.13 MHz, DMSO-*d_6_*): *δ* 10.32 (s, 1H, 7-O*H*), 9.52 (s, 1H, 4′-O*H*), 9.30 (s, 1H, 8-O*H*), 9.28 (s, 1H, 3′-O*H*), 8.97 (s, 1H, 3-O*H*), 7.77 (d, *J* 2.1 Hz, 1H, H-2′), 7.64 (dd, *J* 8.5 and 2.1 Hz, 1H, H-6′), 7.43 (d, *J* 8.7 Hz, 1H, H-5), 6.94 (d, *J* 8.7 Hz, 1H, H-6), 6.89 (d, *J* 8.5 Hz, 1H, H-5′) ppm. ^13^C NMR (75.47 MHz, DMSO-*d_6_*): *δ* 172.4 (C=O), 148.7 (C-7), 147.3 (C-4′), 145.8 (C-9), 145.0 (C-2,3′), 136.9 (C-3), 132.8 (C-8), 122.8 (C-1′), 120.0 (C-6′), 115.5 (C-5′), 115.2 (C-5,10), 115.0 (C-2′), 113.9 (C-6) ppm. ESI-MS *m*/z (%): 341 ([M + K]^+^, 9), 325 ([M + Na]^+^, 34), 303 ([M + H]^+^, 51). EI-HRMS *m*/z calculated for C_15_H_10_O_7_: 302.0427, found: 302.0428.

The ^1^H and ^13^C NMR spectra for melanoxetin are available in the Supplementary Material provided in reference [29].

### 2.2. Chemicals

The following reagents were purchased from Sigma-Aldrich, Inc. (St. Louis, MO, USA): dimethylsulfoxide (DMSO), Tris-HCl, NaCl, sodium dodecyl sulphate (SDS), 2-mercaptoethanol, ethylenediaminetetraacetic acid (EDTA), Triton X-100, and bovine serum albumin (BSA). The molecular weight marker, Prestained Protein Ladder, was acquired from Abcam (Cambridge, UK). A BCA Protein Assay Kit was obtained from BioRad Laboratories (Hercules, CA, USA). The “PGE_2_ Enzyme Immunoassay (EIA) Kit” was acquired from Enzo Life Sciences (Lausen, Switzerland). A list of the primary antibodies employed in this study, along with their respective dilutions, can be found in the Supplementary Material (Supplementary Table S1).

### 2.3. Animal Maintenance and Experimental Design

Four-month-old male Wistar and GK rats from our breeding colonies (Faculty of Medicine, University of Coimbra) were kept under standard conditions (22.0 ± 0.1 °C, 52.0 ± 2.0% relative humidity, 12 h light/dark cycle). All procedures involving animals were previously approved by the local animal welfare commission (ORBEA 13/18) and National Veterinary Authority, following the European Community guidelines for using animals in a laboratory (Directive 2010/63/EU), and performed by users licensed by the Federation for European Laboratory Animal Science Association (FELASA).

**Figure 2:**
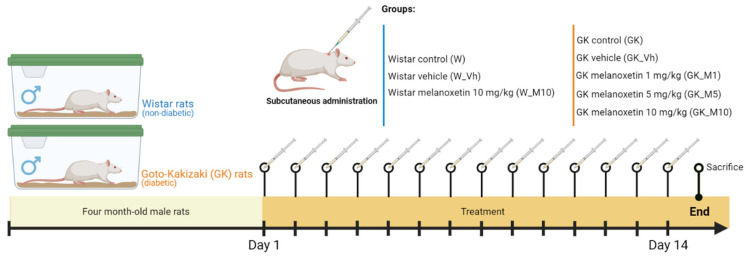
Schematic representation of the in vivo experimental design.

This study aimed to investigate the effects of melanoxetin in male non-diabetic Wistar rats and male GK rats (animal model for type 2 DM). In both male Wistar and male GK rats, the vehicle and melanoxetin were administered subcutaneously in the dorsolumbar region over 14 days. The administration was carried out using a solution containing 50 % DMSO in a volume ranging from 60 and 70 μL, adjusted according to the body weight. Each group of rats studied is outlined below (Figure 2):

- Male non-diabetic Wistar rats:
  ○ Wistar non-diabetic control group (W): This group comprised male Wistar rats without DM, serving as the non-diabetic control group (*n* = 8).
  ○ Wistar vehicle group (W_Vh): Male non-diabetic Wistar rats received treatment with DMSO, serving as the vehicle (*n* = 4).
  ○ Wistar melanoxetin-treated group (W_M10): In this group, male non-diabetic Wistar rats received the highest administered dose of melanoxetin (10 mg/kg) (*n* = 3).
- Male GK rats (Type 2 DM Animal Model):
  ○ GK control group (GK): This group consisted of male GK rats, serving as the diabetic control group (*n* = 6).
  ○ GK vehicle group (GK_Vh): Male GK rats received treatment with DMSO as the vehicle (*n* = 8).
  ○ GK 1 mg/kg melanoxetin group (GK_M1): Male GK rats received a dose of 1 mg/kg of melanoxetin (*n* = 5).
  ○ GK 5 mg/kg melanoxetin group (GK_M5): Male GK rats received a dose of 5 mg/kg of melanoxetin (*n* = 8).
  ○ GK 10 mg/kg melanoxetin group (GK_M10): Male GK rats received the highest dose of melanoxetin (10 mg/kg) (*n* = 6).

### 2.4. In Vivo Procedures and Sample Collection

Body weight as well as caloric and water intake were monitored during the treatment. Fasting blood glucose (measured after a 6 h fast) was determined in blood samples collected from the tail vein on the final day of the in vivo experiment. An intraperitoneal insulin tolerance test (ITT) was performed after a 6 h fast, using insulin per body weight (250 mU/kg) and the evaluation of glycaemia was performed at 0, 15, 30 and 60 min using a glucometer and test strips (Accu-Chek Aviva, Roche, Basel, Switzerland). Animals were anesthetized with an intraperitoneal injection of 2:1 (*v*/*v*) 50 mg/kg ketamine (100 mg/mL)/2.5% chlorpromazine (5 mg/mL). Blood was collected through cardiac punctures to Vacuette K_3_EDTA tubes (Greiner Bio-One, Kremsnunster, Austria) and centrifuged for plasma collection. The plasma was used for the measurement of plasma insulin levels using a Rat Insulin ELISA Kit (Mercodia, Uppsala, Sweden) according to the manufacturer’s instructions. After the blood sample collection, animals were sacrificed via cervical displacement, and the following tissues were collected: aorta, epididymal adipose tissue (EAT), pancreas, liver and heart. All tissues were stored at −80 °C for western blotting analysis.

### 2.5. Functional Studies of Aorta

Aorta rings were mounted on stainless-steel hooks in organ baths (basal tension = 19.6 mN) filled with aerated Krebs-Henseleit solution (NaCl 118.67 mM; KCl 5.36 mM; CaCl_2_ 1.90 mM; MgSO_4_ 0.57 mM; NaHCO_3_ 25.00 mM; KH_2_PO_4_·H_2_O 0.90 mM; glucose 11.1 mM; 37 °C, pH 7.4; 95% O_2_, 5% CO_2_). After a 60 min equilibration period, cumulative isometric concentration–response curves to acetylcholine (ACh) (0.01 to 90 µM) were performed in a noradrenaline-precontracted aorta ring (10 µM) in the presence and absence of 100 µM ascorbic acid and recorded with MLT050/D isometric force transducers (Panlab, Barcelona, Spain) coupled to a bridge amplifier ML224 and PowerLab 4/30 data acquisition system (ADInstruments, Oxford, UK).

### 2.6. Western Blotting

The EAT, heart, liver and aorta (*n* = 4–6 per group) were homogenized with lysis buffer [0.25 M Tris-HCl, 125 mM NaCl, 1% Triton X-100, 0.5% SDS, 1 mM EDTA, 20 mM NaF, 2 mM Na_3_VO_4,_ 10 mM β-glycerophosphate, 2.5 mM of sodium pyrophosphate, 10 mM of PMSF, and 40 µL of protease inhibitor, used according to the manufacturer’s instructions (Roche, Germany)]. Lysates were centrifuged and the supernatant fractions were used for protein quantification using the BCA Protein Assay Kit. Total protein (15–50 µg) was prepared using Laemmeli buffer (62.5 mM Tris-HCl, 10% glycerol, 2% SDS, 5% β-mercaptoethanol, 0.01% bromophenol blue). Samples were loaded onto 8–10% polyacrylamide gel, separated through electrophoresis, and transferred to polyvinylidene fluoride (PVDF) membranes. After blocking (TBS-T 0.01% and 5% BSA, or 5% *w*/*v* nonfat dry milk), membranes were incubated overnight at 4 °C with primary antibodies, followed by a 2 h incubation at room temperature with secondary antibodies. Immunoblot detection occurred using an enhanced chemiluminescence (ECL) substrate in the LAS 500 system (GE-Healthcare, Chicago, IL, USA). The bands were quantified with Image Quant software 7.0 (Molecular Dynamics, Sunnyvale, CA, USA). The presented values are expressed as percentages following normalization with the Wistar group serving as the reference point set at 100%, and normalized for the respective loading control (calnexin, 83 kDa or GADPH, 37 kDa, or β-actin, 42 kDa).

### 2.7. Histological Analysis

The pancreas was fixed with 10% formalin solution, dehydrated in crescent alcohol concentrations (70%, 95% and 100%), cleared in xylene, and embedded in paraffin. The tissue was sectioned in a microtome, on a non-serial section that was 4 µm thickness (*n* = 3–4/group) and subsequently dried overnight at room temperature. The tissue sections were stained via hematoxylin and eosin (H&E) staining. Images were obtained using a microscope (Zeiss Axio Observer Z1, Carl Zeiss, Gottingen, Germany).

### 2.8. Determination of PGE_2_ Production

A PGE_2_ ELISA kit was used to determine the amount of PGE_2_ in the serum samples of all groups, according to the manufacturer’s instructions. The results were expressed as pg/mL of PGE_2_ concentration.

### 2.9. Statistical Analysis

The results were expressed as the mean ± standard error of the mean (SEM) per group. A statistical analysis was performed using GraphPad Prism 9 (GraphPad Software, San Diego, CA, USA). The normality of the data was assessed with a Shapiro–Wilk normality test. According to the test results, for the parametric data, a parametric one-way Analysis of Variance (ANOVA) was applied, followed by Tukey’s multiples comparison test. For the non-parametric data, the Kruskal–Wallis test was used. A *p*-value < 0.05 was considered statistically significant.

## 3. Results

### 3.1. Melanotexin Reduces Body Weight in Normal Rats While Exhibiting No Alterations in Glycemia, Insulin Levels, or Pancreatic Islet Morphology in Diabetic Rats

**Figure 3:**
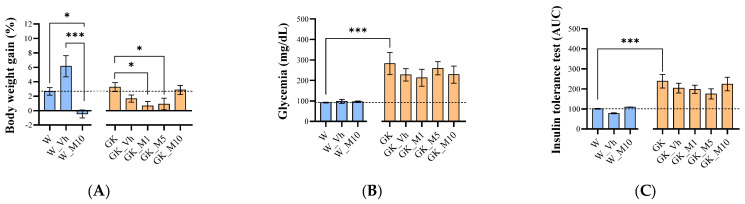
Effects of melanoxetin on body weight gain (**A**), glycemia (**B**), and insulin tolerance test (**C**) in normal and diabetic animals. Results are expressed as the mean ± SEM of three to six animals per group. * *p* < 0.05, *** *p* < 0.001.

The effects of melanoxetin on general morphology and welfare were evaluated across several parameters, including body weight, caloric intake, water intake, EAT weight, insulin levels, and glycemia. Examining the effects of melanoxetin on body weight gain, the results show distinct outcomes within both Wistar non-diabetic and GK diabetic rats. In Wistar control animals, the administration of melanoxetin at a dose of 10 mg/kg (W_M10) led to a significant decrease in body weight gain compared with the control (W) (*p* < 0.05) and vehicle (W_Vh) (*p* < 0.001) groups (Figure 3A) at 14 days. In diabetic animals, the administration of melanoxetin at 1 mg/kg and 5 mg/kg (GK_M1 and GK_M5) resulted in a significant reduction of body weight gain compared with the control group (GK) (*p* < 0.05), but not the vehicle group, as depicted in Figure 3A.

No significant differences were observed in glycemia (Figure 3B), insulin tolerance (Figure 3C), caloric intake, water intake levels, or EAT weight following melanoxetin administration in both Wistar and GK rats (Supplementary Figure S1).

**Figure 4:**
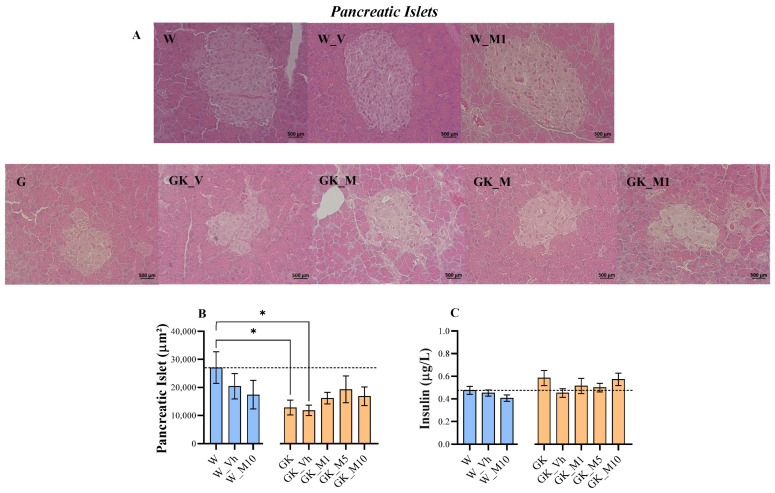
Representative microphotographs (scale bar = 500 µm, magnification = 400×) of pancreatic islets after hematoxylin–eosin staining (**A**), graphical representation of pancreatic islet area (**B**), and plasma insulin levels (**C**). Results are expressed as the mean ± SEM of three to six animals per group. * *p* < 0.05.

The histological analysis of pancreatic islets showed that the GK and GK_Vh groups had significantly smaller pancreatic islet areas compared with the W group (*p* < 0.05). No differences were observed after melanoxetin administration in both Wistar control and GK rats (Figure 4A,B). In addition, the levels of plasma insulin (Figure 4C) did not show significant differences following melanoxetin administration.

### 3.2. Melanoxetin Induces Changes in PPARγ and PTP1B Expression within Epididymal Adipose Tissue

The effects of the flavonoid melanoxetin were assessed on the insulin signaling pathway in the EAT, encompassing the insulin receptor (IR), total AMP-activated protein kinase (AMPK), *p*-AMPK, glucose transporter protein type 4 (GLUT4), peroxisome proliferator-activated receptor gamma (PPARγ), and protein tyrosine phosphatase 1B (PTP1B), as well as in the liver involving the IR, total AMPK, *p*-AMPK, glucose transporter protein type 2 (GLUT2), peroxisome proliferator-activated receptor alpha (PPARα), PTP1B, and fructose 1,6-bisphosphatase (FBPase).

**Figure 5:**
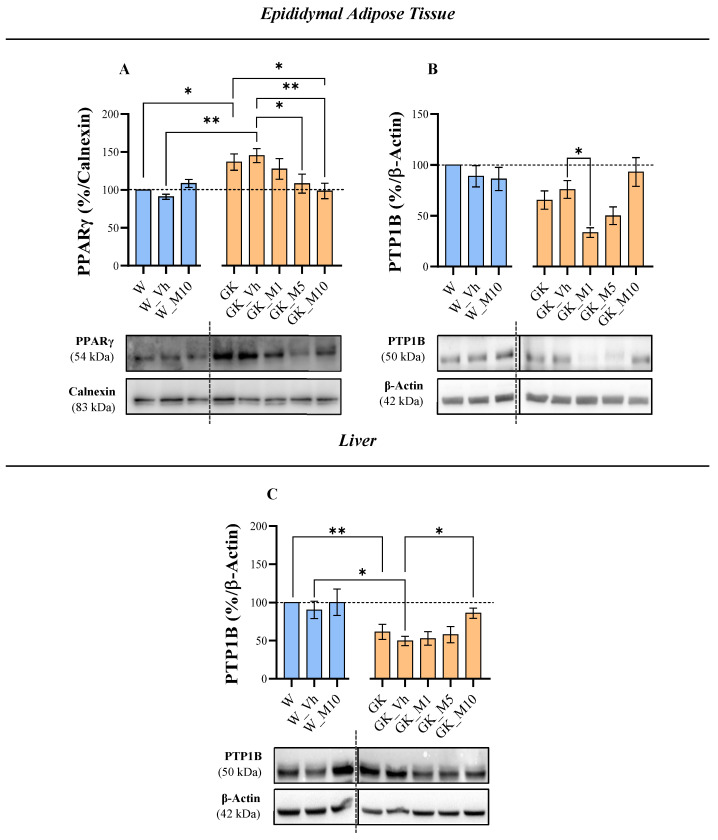
Effects of melanoxetin on the expression of PPARγ (**A**) and PTP1B (**B**) in epididymal adipose tissue (EAT) and PTP1B (**C**) expression in the liver. Results are expressed as the mean ± SEM of three to six animals per group. * *p* < 0.05, ** *p* < 0.01. Full pictures of the Western blots and the densitometry scans are presented in File S1.

In relation to insulin signaling in the EAT, no alterations were observed following melanoxetin administration in the IR, total AMPK, *p*-AMPK, and GLUT4 when compared to the GK_Vh group (Supplementary Figure S2). The administration of melanoxetin induced alterations in the levels of PPARγ and PTP1B. In particular, reductions in PPARγ were observed at doses M5 and M10 compared to the GK_Vh group (*p* < 0.05 and *p* < 0.01, respectively), normalizing the PPARγ values of GK animals to those observed in Wistar control rats (Figure 5A). In what concerns the levels of PTP1B in EAT, a hormesis response was observed in the GK treated animals, where the GK_M1 group showed a significant decrease in PTP1B levels compared to the GK_Vh group (*p* < 0.05) (Figure 5B), which was abrogated at higher doses.

Regarding insulin signaling in the liver tissue, no differences were detected with melanoxetin in the levels of the IR, total AMPK and its phosphorylation, GLUT2, PPARα and FBPase, compared to the GK_Vh group (Supplementary Figure S3). Concerning PTP1B levels, an increase in PTP1B levels in the GK_M10 group was observed, when compared to the GK_Vh group (Figure 5C).

### 3.3. Melanoxetin Modulates Oxidative Stress and the Expression of Antioxidant Enzymes in the Epididymal Adipose and Liver Tissues of Diabetic Rats

To assess the effects of the flavonoid melanoxetin on oxidative stress, the expression of the following parameters was evaluated: catalase, glyoxalase 1 (GLO1), nuclear factor erythroid 2-related factor 2 (Nrf2), superoxide dismutase type 1 (SOD1), hemeoxygenase, methylglyoxal-derived hydroimidazolone (MG-H1), argpyrimidine (Arg-P), nitrotyrosine in EAT; whereas catalase, GLO1, Nrf2, SOD1, hemeoxygenase, and MG-H1 was assessed in liver.

**Figure 6:**
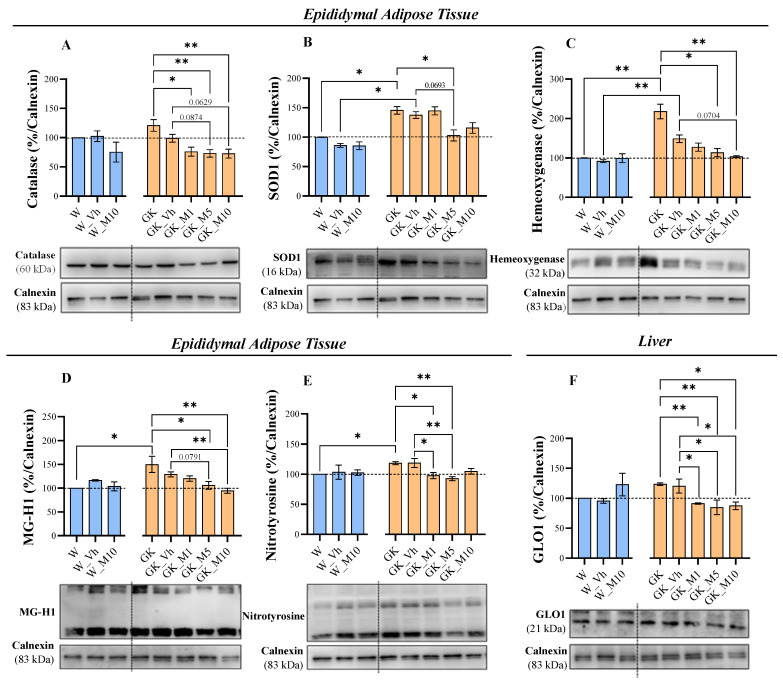
Melanoxetin modulates antioxidant enzymes and a marker of nitration in EAT, depending on drug concentrations, avoiding the accumulation of AGEs in type 2 DM animals: catalase (**A**), SOD1 (**B**), hemeoxygenase (**C**), MG-H1 (**D**), and nitrotyrosine (**E**). Melanoxetin reduces the expression of GLO1 in the liver (**F**). Results are expressed as the mean ± SEM of three to six animals per group. * *p* < 0.05, ** *p* < 0.01.

In EAT, the GK group presented significantly higher levels of SOD1 (*p* < 0.05), hemeoxygenase (*p* < 0.01), MG-H1 (*p* < 0.05), and nitrotyrosine (*p* < 0.05), in comparison to the W control animals (Figure 6). Melanoxetin administration showed differences in GK diabetic animals by reducing the levels of the antioxidant enzymes catalase, SOD1, and hemeoxygenase, when compared to the GK_Vh group, but not in Wistar animals (Figure 6). Interestingly, at 5 and 10 mg/kg of melanoxetin administration in GK rats, there was a tendency for reductions in catalase levels compared with the GK_Vh group (*p* = 0.0874 and *p* = 0.0629, respectively) (Figure 6A). The GK animals receiving 5 mg/kg also tended to show a decrease in the levels of the antioxidant enzyme SOD1, when compared to the GK_Vh group, with *p* = 0.0693 (Figure 6B). Regarding the hemeoxygenase levels, a tendency for reduction was also observed in the GK rats receiving 10 mg/kg, when compared to the GK vehicle group (*p* = 0.0704) (Figure 6C). The administration of melanoxetin also resulted in a decrease in the AGE MG-H1, as well as a reduction in the oxidative stress biomarker nitrotyrosine in EAT (Figure 6). A reduction was observed in the MG-H1 levels with the administrations of 5 and 10 mg/kg melanoxetin in GK animals when compared with the GK_Vh group (being that in the lower concentration, a tendency was found *p* = 0.0791, and in the higher concentration, a statistical significance was found *p* < 0.01) (Figure 6D). Concerning the nitrotyrosine levels, significant reductions were noted for 1 and 5 mg/kg melanoxetin (*p* < 0.05 and *p* < 0.01, respectively) in GK animals, compared to the GK_Vh group (Figure 6E). The expression of GLO1, Nrf2, and Arg-P was not significantly altered in the GK diabetic animals following the administration of the flavonoid melanoxetin (Supplementary Figure S4).

Regarding the expression of oxidative stress and antioxidant enzymes in the liver, only GLO1 levels showed significant differences after melanoxetin administration, with significantly reduced levels at different concentrations—GK_M1 (*p* < 0.01 and *p* < 0.05), GK_M5 (*p* < 0.01 and *p* < 0.05), and GK_M10 (*p* < 0.05)—in comparison with the GK and GK_Vh groups (Figure 6F). The expression levels of catalase, Nrf2, SOD1, hemeoxygenase, and MG-H1 were not altered (Supplementary Figure S5).

### 3.4. Melanoxetin Reduces Cardiovascular Complications Associated with Diabetes by Reducing Oxidative Stress in the Heart and Increasing Acetylcholine-Dependent Vasorelaxation in the Aorta

**Figure 7:**
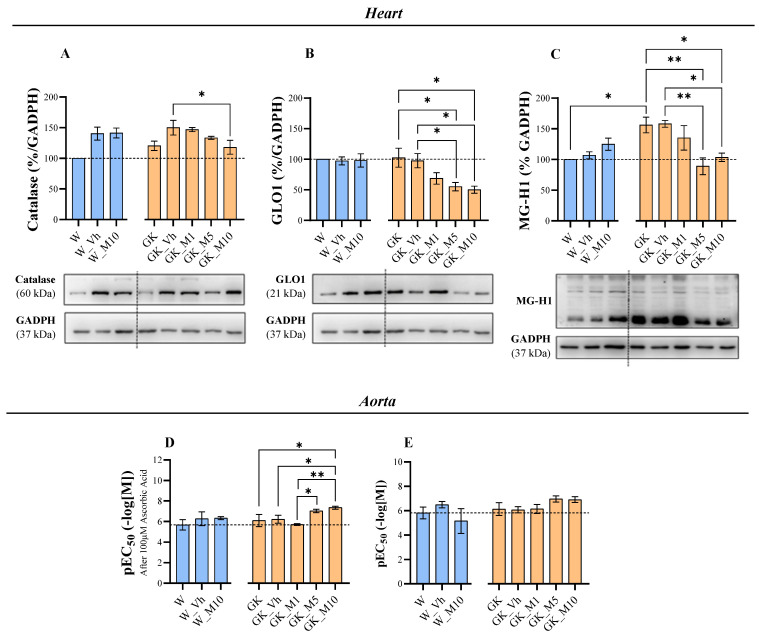
Melanoxetin reduces the expressions of catalase (**A**), GLO1 (**B**), and MG-H1 (**C**) in the heart. Melanoxetin increases ACh-dependent relaxation in the presence of ascorbic acid (**D**), having no effects in the absence of ascorbic acid (**E**). Results are expressed as the mean ± SEM of three to six animals per group. * *p* < 0.05, ** *p* < 0.01.

In the heart, the expression levels of catalase (Figure 7A), GLO1 (Figure 7B), and MG-H1 (Figure 7C) were significantly altered with the administration of melanoxetin. The treatment with melanoxetin in the highest concentrations (5 mg/kg and 10 mg/kg) significantly reduced the levels of GLO1 and MG-H1, when compared to the GK_Vh groups. Additionally, at the 10 mg/kg dose, the levels of catalase were also significantly reduced. No significant alterations were observed in the Nrf2, SOD1, hemeoxygenase, and nitrotyrosine expressions (Supplementary Figure S6).

In the aorta, the expressions of catalase (Supplementary Figure S7A) and GLO1 (Supplementary Figure S7B) were also assessed, showing no statistically significant differences after melanoxetin administration.

Aortic relaxation in response to ACh was evaluated as a measure of vascular function, and the response to the antioxidant ascorbic acid was employed as a marker of the antioxidant status of the vessel wall. Dose–response curves were generated for cumulative concentrations of ACh, in the absence and presence of ascorbic acid (Supplementary Figure S8). At this age, the diabetic rats did not exhibit alterations in ACh-dependent relaxation, although it is known that they develop endothelial dysfunction over time [30]. Melanoxetin did not change the basal response to ACh (Figure 7E), as measured using the maximum response (Emax) and negative logarithm of the EC_50_ (pEC_50_). However, it potentiated the response in the presence of ascorbic acid (Figure 7D), indicating a higher pEC_50_ and suggesting an improved antioxidant profile, which is essential for ^●^NO bioavailability. The Emax remained unchanged.

### 3.5. Melanoxetin Induces the Hormetic Suppression of Serum PGE_2_ Levels in Diabetic Rats

**Figure 8:**
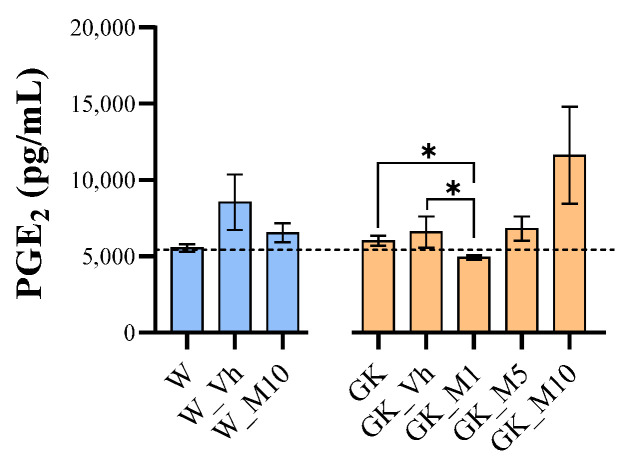
Melanoxetin avoids the production of PGE_2_ in the serum of type 2 DM animals with a hormesis-type effect. Results are expressed as the mean ± SEM of three to six animals per group. * *p* < 0.05.

The production of PGE_2_ was assessed in the serum of both the normal and diabetic groups to evaluate the anti-inflammatory effects of melanoxetin (Figure 8). No significant differences in PGE_2_ production were observed in normal animals following melanoxetin administration. However, in diabetic animals, melanoxetin administration exhibited a hormesis-type effect, with a significant reduction in PGE_2_ production in the GK_M1 group, when compared with the GK (*p* < 0.05) and GK_Vh groups (*p* < 0.05).

## 4. Discussion

Studies on both humans and animals have shown that certain polyphenols can reduce oxidative stress and hyperglycemia, as well as prevent diabetic complications [31]. This study elucidates the antidiabetic, antioxidant, and anti-inflammatory properties of the flavonoid melanoxetin, using GK rats, an animal model of type 2 DM. GK rats represent a non-obese model of type 2 DM and exhibit insulin resistance and an insulin secretory defect [32]. To assess the effectiveness of the studied flavonoid, subcutaneous administration was employed with varying doses (1, 5, and 10 mg/kg) over a 14-day treatment period. It is worth noting that this study represents a preliminary investigation into the in vivo effects of melanoxetin.

In the present study, melanoxetin administration was not sufficient to effectively reduce hyperglycemia and hyperinsulinemia in GK diabetic animals. However, interesting effects were seen: a significant decrease in the body weight gain in normal animals and a tendency to reduce body weight in diabetic animals, particularly with a hormesis effect in GK diabetic animals, after melanoxetin administration for 14 days. Since GK rats are a lean model of DM and are smaller than control Wistar rats, one less significant difference was already expected. This characteristic can have a beneficial impact on type 2 DM, particularly considering that the majority of DM patients are overweight or obese. In fact, DM and obesity are considered the twin epidemics of the 21st century. It is noteworthy that obesity is associated with an elevated risk of various diseases, such as DM, hypertension, NAFLD, and CVD. Addressing obesity presents a significant challenge, as the range of available medications is currently restricted. Metreleptin and setmelanotide are prescribed for rare obesity syndromes, and orlistat, phentermine/topiramate, naltrexone/bupropion, liraglutide, and semaglutide for non-syndromic obesity. However, these medications come with various adverse effects [33]. Hence, discovering compounds capable of inducing weight loss could be a promising approach to addressing DM.

The insulin signaling pathway was studied in EAT and in liver tissue. The insulin cascade is initiated by the binding of insulin to its receptor, culminating in the activation of glucose transporters, GLUT4 in EAT, and GLUT2 in the liver, to enable the transport of glucose inside the cell [5]. In this study, the administration of melanoxetin had no significant effects on the expressions of IR, total AMPK, *p*-AMPK, GLUT2, and GLUT4 in EAT and liver tissue. Interestingly, we observed that the administration of melanoxetin could modulate PTP1B levels in the EAT of diabetic animals, showing significant results, particularly with the lower administered dose (1 mg/kg). It is widely recognized that insulin resistance is a crucial metabolic dysfunction commonly linked to type 2 DM, referring to the impaired ability of peripheral target tissues, including adipose tissue, to effectively respond to insulin. PTP1B is a target under investigation, identified as a key negative regulator of the insulin signaling pathway, able to dephosphorylate IR and insulin receptor substrates [34]. PTP1B is also a negative regulator of leptin signaling, which is an important hormone for the regulation of food intake, body weight, proinflammatory immune responses, angiogenesis, and lipolysis. Its actions, such as insulin effects, are impaired in obesity. Therefore, the reduction of PTP1B would be an important target in type 2 DM and obesity [35]. Until now, few drugs have reached clinical trials, including ertiprotafib, trodusquemine, and JTT-551, targeting PTP1B. However, inhibitors of PTP1B are not yet currently approved for clinical use [36]. As far as we know, this is the first study showing the effect of melanoxetin on PTP1B levels.

PPARγ levels were also assessed in EAT. Our results revealed that GK rats present a heightened expression of PPARγ, when compared to normal Wistar rats. The enhanced PPARγ expression in GK rats may present itself as an adaptive mechanism for adipose tissue dysfunction in diabetic conditions. The administration of melanoxetin normalized PPARγ levels at higher doses, restoring them to normal values, mirroring those observed in normal Wistar rats. This normalization suggests a potential role for melanoxetin in mitigating the adipose tissue dysfunction associated with DM. Concerning PPARα, primarily abundant in the liver, melanoxetin administration did not yield significant alterations. This finding underscores a possible specificity of melanoxetin’s impact on the PPAR expression, with a pronounced effect observed in PPARγ within adipose tissue. To the best of our knowledge, this study represents the first assessment of the impact of melanoxetin on the expression levels of PPARs.

To comprehensively evaluate the influence of the flavonoid melanoxetin on oxidative stress, we assessed the expression of a spectrum of parameters in EAT and in the liver. Significantly, the most pronounced effects were observed in the EAT, where notable modulations of the antioxidant enzymes and oxidative stress markers were evident. In the liver, fewer alterations were observed in the oxidative stress markers. Adipose tissue is highly active in regulating metabolic processes and can be more susceptible to changes, compared to the liver [37]. Therefore, the differences observed between the effect of the compound in the liver and adipose tissue of GK rats might be due to tissue-specific responses.

Oxidative stress can be defined as an imbalance between the excessive production of reactive pro-oxidant species and the capacity of the existing antioxidant defense mechanisms to counteract them. The overproduction of reactive pro-oxidant species in the diabetic environment results from hyperglycemia, attributed to the increase in AGEs and other mediators, such as cytokines. The overproduction of reactive pro-oxidant species can directly induce structural and functional modifications in proteins, lipids, and nucleic acids. Furthermore, it modulates intracellular signaling pathways that contribute to insulin resistance and the impairment of β cell function [38]. The excessive production of reactive pro-oxidant species should induce the expression and activity of antioxidant enzymes as a compensatory mechanism, upregulating the antioxidant defense mechanisms, primarily through enzymatic scavengers like SOD and catalase [39]. Therefore, in this study, the evaluation of key antioxidant enzymes was driven by their crucial roles in counteracting the excessive production of reactive pro-oxidant species. The compensatory upregulation of antioxidant defense mechanisms, particularly through enzymatic scavengers like SOD and catalase, serves as a vital cellular response to mitigate oxidative stress. In addition, increased levels of nitrotyrosine are also found in patients with type 2 DM [40]. Peroxynitrite is a potent oxidant and nitrating agent that is associated with adverse effects, including the reduction of antioxidant defenses and the inactivation of important enzymes. Nitrotyrosine is a product of peroxynitrite action and has been demonstrated to have a direct relation involving postprandial hyperglycemia and the production of nitrotyrosine, being a possible marker of oxidative/nitrosative stress in type 2 DM patients [41]. Consistent with previous findings, we observed a notable increase in antioxidant defense markers, including SOD1 and hemeoxygenase, and also nitrotyrosine, a marker of nitration, in type 2 DM GK rats compared to non-diabetic Wistar rats. Notably, melanoxetin administration changed those values to those similar to the observed in normal Wistar rats.

Persistent hyperglycemia in type 2 DM leads to the development of a non-enzymatic glycation reaction with protein, lipids, and nucleic acids, culminating in the formation of several molecules known as AGEs. This group of molecules is recognized by its actions in the pathophysiology of diabetic complications, due to increased oxidative stress [42]. Methylglyoxal (MG), a glucose-derived reactive metabolite formed primarily as a byproduct of glycolysis, is a major precursor of AGEs. Despite the lower concentration of MG in tissues, when compared with glucose, MG exhibits approximately 20,000 times higher reactivity than glucose. The molecule MG-H1 is a key derivative of MG and is recognized as a major AGE in DM [43,44]. The primary physiological mechanism for detoxifying MG is the glutathione (GSH)-dependent glyoxalase (GLO) system, which comprises GLO1, GLO2, and reduced GSH [43,44]. Our results demonstrate a significant decrease in the expression of GLO1 in the liver following melanoxetin administration. In addition to these results, a decrease in the MG-H1 expression was observed after melanoxetin administration in EAT.

In light of the interrelation between DM and CVD, the effects of the compound melanoxetin on oxidative stress markers within the heart and aorta tissue were evaluated, as well as the vasorelaxation of the aorta. Reductions in the expressions of the catalase, MG-H1, and GLO1 levels were found in the heart tissue following melanoxetin administration in GK animals. This decline in GLO1 expression is notably interesting as it correlates with the reduction in AGEs, particularly MG-H1 in the heart, as observed in our study.

In the aorta, catalase and GLO1 expressions were also evaluated, indicating no statistically significant differences following melanoxetin administration. However, these results do not provide a comprehensive understanding of the redox status of the vessel wall. ^●^NO availability is strongly dependent on the redox status of the vessel wall. In this context, ACh-induced relaxation in basal conditions and in the presence of ascorbic acid was evaluated. These results serve as an indicator of the redox status, as they reflect the availability of ^●^NO in the vessel wall in the presence of an antioxidant. The diabetic rats treated with higher doses of melanoxetin demonstrated higher responses to ACh in the aortic relaxation in the presence of the antioxidant ascorbic acid. These observations suggest an improvement in the redox status of the vessel wall. Moreover, since ACh-induced relaxation is dependent on ^●^NO, the enhanced relaxation observed might imply increases in the ^●^NO concentrations in these animals. This could be correlated with the decreased levels of nitrotyrosine within GK rats, suggesting a possibly improved endothelial function due to the reduced oxidative stress.

In observing the results from the heart and aorta, it suggests that melanoxetin may be exerting its effects by directly reducing oxidative stress and the formation of AGEs, resulting in the reduction of the compensatory increase in the GLO system. Since this system is GSH-dependent, its upregulation in disease conditions contributes to GSH depletion and its normalization allows GSH restoration.

As far as we know, this is the first in vivo study concerning the use of melanoxetin as an antioxidant agent across several markers of oxidative stress and AGEs. Bodede et al. [28] already studied melanoxetin and other related flavonoids in the ROS production in RAW264.7 cells and found that melanoxetin was able to attenuate the lipopolysaccharide (LPS)-induced intracellular ROS production in this cellular model [28], but no other meaningful study has been conducted.

DM is also associated with the presence of chronic tissue inflammation, such as in adipose tissue, liver, muscle, and pancreatic islets. Inflammation in DM is related to the progression of several complications [45]. PGE_2_, an inflammatory mediator, is an arachidonic acid metabolite, which is formed by several enzymatic reactions, mainly by the action of COX-1 or COX-2. PGE_2_ is one of the most highly expressed prostaglandins by nearly all cell types within the body. In animal models of type 2 DM and in type 2 DM patients, the precursors of arachidonic acid and its metabolites have been demonstrated to be increased in biological fluids, including serum and urine. Therefore, some studies suggest using PGE_2_ as a biomarker of type 2 DM and also as a therapeutic target. It is noteworthy that hyperglycemia, dyslipidemia, and pro-inflammatory cytokines have all demonstrated the capacity to upregulate enzymes within the PGE_2_ production pathway [46]. Furthermore, the increase in PGE_2_ levels may also be associated with an augmented state of oxidative stress [47]. In addition, some studies indicate that the use of antioxidants could reduce the expression levels in COX-2, thereby reducing the PGE_2_ levels [48]. ln our study, we measured the levels of PGE_2_ in the serum of both normal and diabetic rats. The results showed no differences between W and GK animals, but the administration of melanoxetin demonstrated a reduction in PGE_2_ levels at the lower dose. These findings align with the study conducted by Wu et al. [25], which examined the effects of melanoxetin in a cellular model, demonstrating a decrease in PGE_2_ levels. However, as far as we know, there have been no published in vivo studies on DM models that have investigated melanoxetin in the serum of diabetic animals.

## 5. Conclusions

In conclusion, our findings reveal that melanoxetin treatment leads to a significant decrease in body weight gain in normal animals, and a reduction in the PTP1B and PPARγ expressions in the EAT of diabetic animals. Notably, the most significant effects of melanoxetin were observed in the EAT, through the modulation of antioxidant enzymes and oxidative stress markers, and in a reduction in MG-H1, a major AGE product, further contributing to the mitigation of oxidative stress. A decrease in the GLO1 expression was also observed in both liver and heart tissues, as well as a better oxidative status of the aortic wall. The administration of melanoxetin also improved DM-related inflammation by reducing PGE_2_ levels in diabetic animals. These findings suggest that melanoxetin holds potential as a therapeutic agent for DM, mainly as a promising antioxidant compound. Further research is needed to investigate the precise underlying molecular mechanisms and long-term effects of melanoxetin treatment.

## Data Availability

The raw data supporting the conclusions of this article will be made available by the authors on request.

## Supplementary Materials

The following supporting information can be downloaded at: https://www.mdpi.com/article/10.3390/pharmaceutics16020261/s1, Figure S1: Effect of melanoxetin on caloric intake (A), water intake (B) EAT weight (C) and insulin tolerance test (D) in normal and diabetic animals; Figure S2: Effect of melanoxetin on the expression of total IR (A), GLUT4 (B), total AMPK (C), and *p*-AMPK (D) in epididymal adipose tissue; Figure S3: Effect of melanoxetin on the expression of Total IR (A), Total AMPK (B), *p*-AMPK (C), GLUT2 (D), PPARα (E) and FBPase (F) in liver tissue; Figure S4: Effect of melanoxetin on the expression of GLO1 (A), Nrf2 (B) and Arg-P (C) in epididymal adipose tissue; Figure S5: Effect of melanoxetin on the expression of catalase (A), SOD1 (B), Nrf2 (C), hemeoxygenase (D), and MG-H1 (E) in liver tissue; Figure S6: Effect of melanoxetin on the expression of SOD1 (A), Nrf2 (B), hemeoxygenase (C), and nitrotyrosine (E) in the heart; Figure S7: Effect of melanoxetin on the expression of GLO1 (A) and catalase (B) in the aorta; Figure S8: Effects of melanoxetin treatment on relaxation response of the isolated to acetylcholine (ACh); Table S1: Table detailing antibody names, dilutions, and respective purchase sources for Western Blot analyses. File S1: Original Western Blot figures.
