# Supplementary material for: Melanoxetin: A Hydroxylated Flavonoid Attenuates Oxidative Stress and Modulates Insulin Resistance and Glycation Pathways in an Animal Model of Type 2 Diabetes Mellitus
Source: Pharmaceutics. 2024 Feb 9;16(2):261. doi: 10.3390/pharmaceutics16020261 (PMC10892797; doi:10.3390/pharmaceutics16020261)
Supplement: Supplementary file 1 [file pharmaceutics-16-00261-s001.zip › pharmaceutics-2836090-File S1.pdf]

Melanoxetin: A Hydroxylated Flavonoid Reduces Oxidative Stress in an Animal Model of Type 2 Diabetes Mellitus

Sónia Rocha, Andreia Amaro, Marcos D. Ferreira-Junior, Carina Proença, Artur M.S. Silva, Vera M. Costa, Sara Oliveira, Diogo A. Fonseca, Sónia Silva, M. Luísa Corvo, Marisa Freitas, Paulo Matafome, Eduarda Fernandes

Original Images of Western Blots

Epididymal Adipose Tissue (EAT)

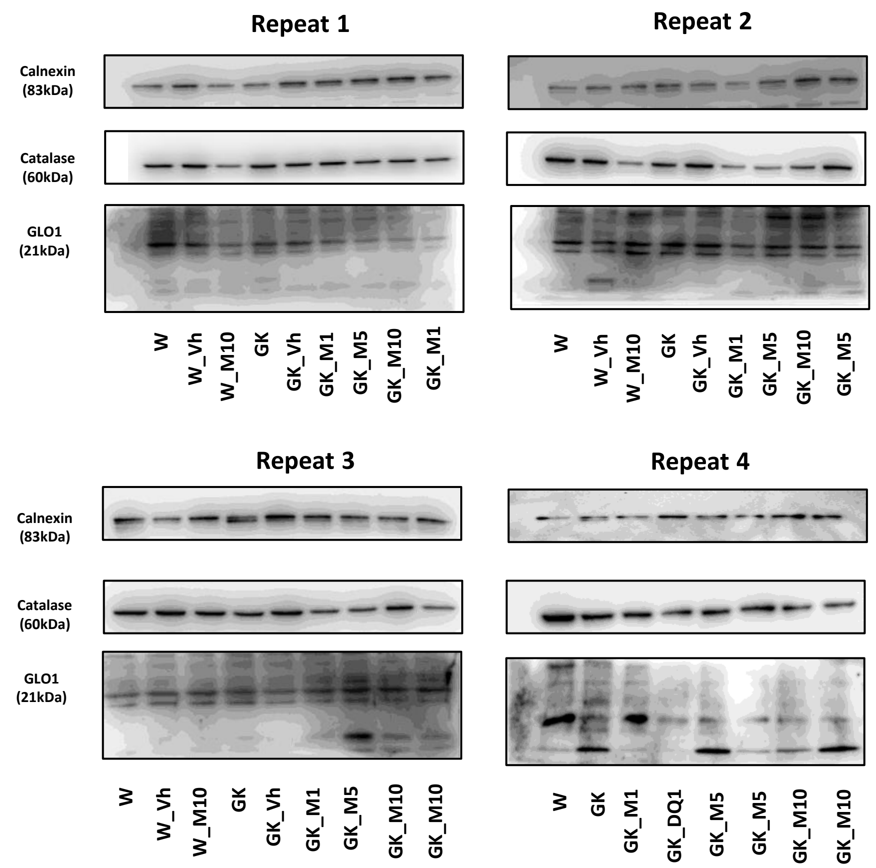

GLO1 [Supplementary figure S4A]
Catalase [Figure 6A]

Epididymal Adipose Tissue (EAT)

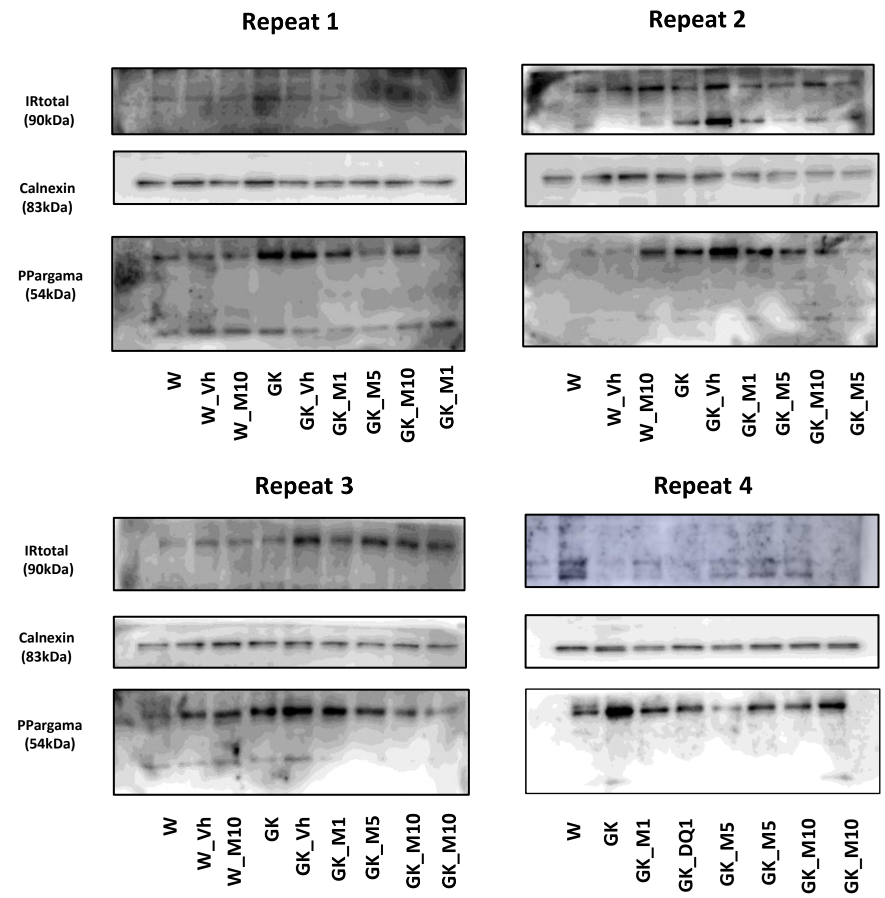

IRtotal [Supplementary figure S2A]
PPARγ [Figure 5A]

Epididymal Adipose Tissue (EAT)

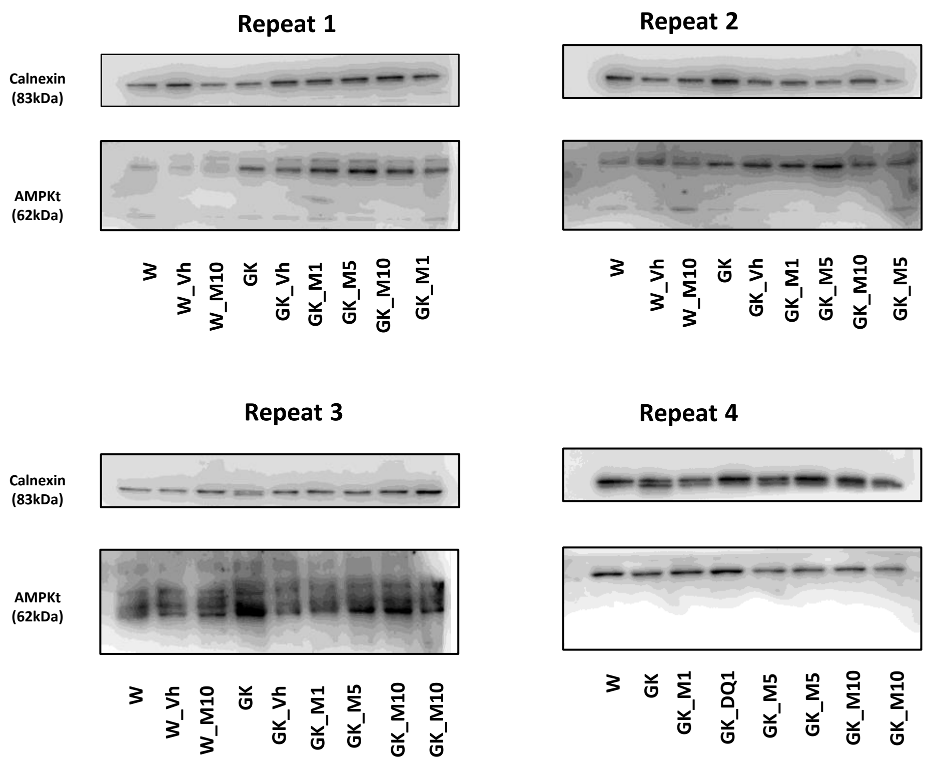

AMPK [Supplementary figure S2C]

Epididymal Adipose Tissue (EAT)

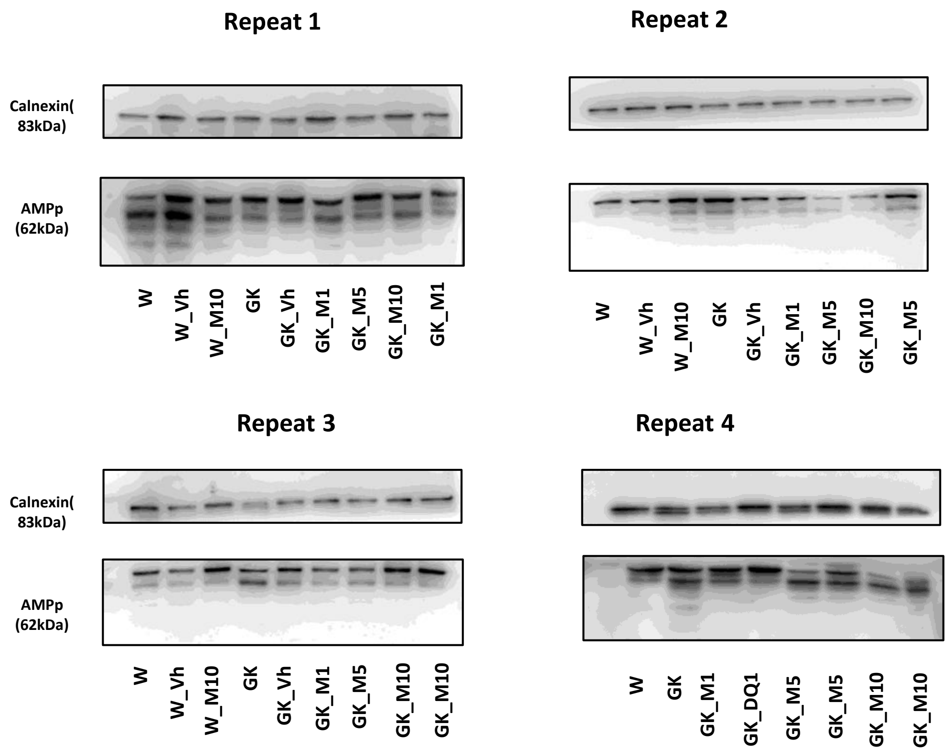

AMPKp [Supplementary figure S2]

Epididymal Adipose Tissue (EAT)

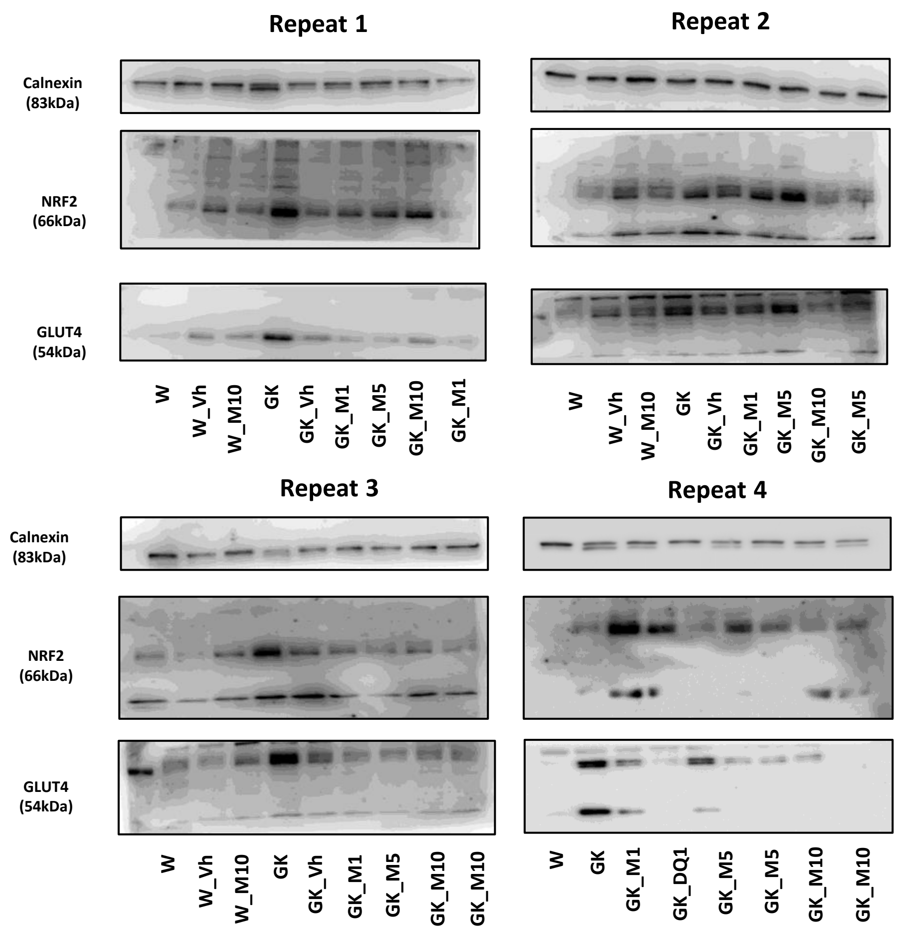

GLUT4 [Supplementary figure S2B]
NRF2 [Supplementary figure S4B]

Epididymal Adipose Tissue (EAT)

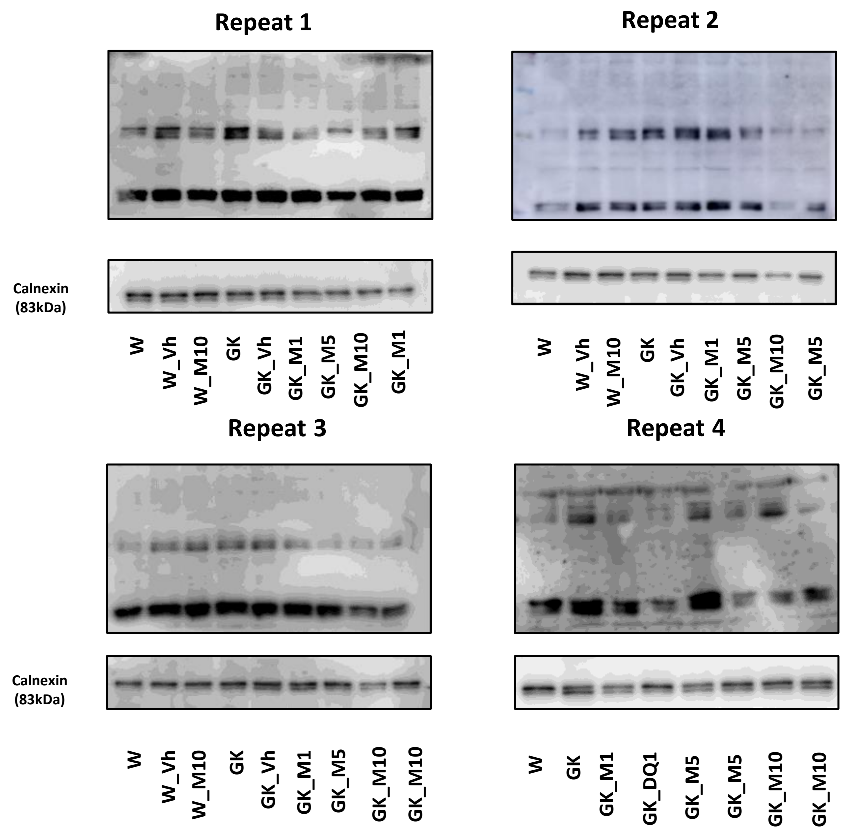

MGH1 [Figure 6D]

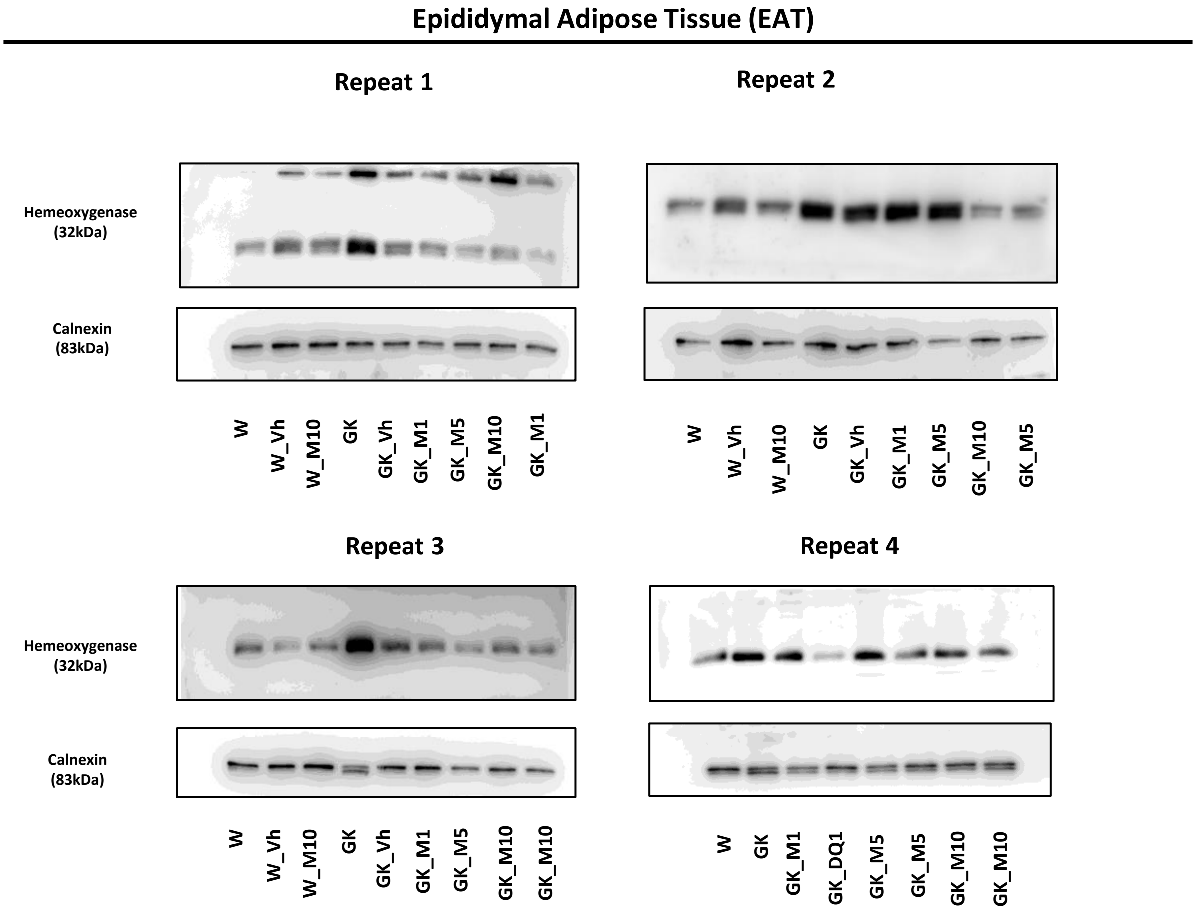

Hemeoxygenase [Figure 6C]

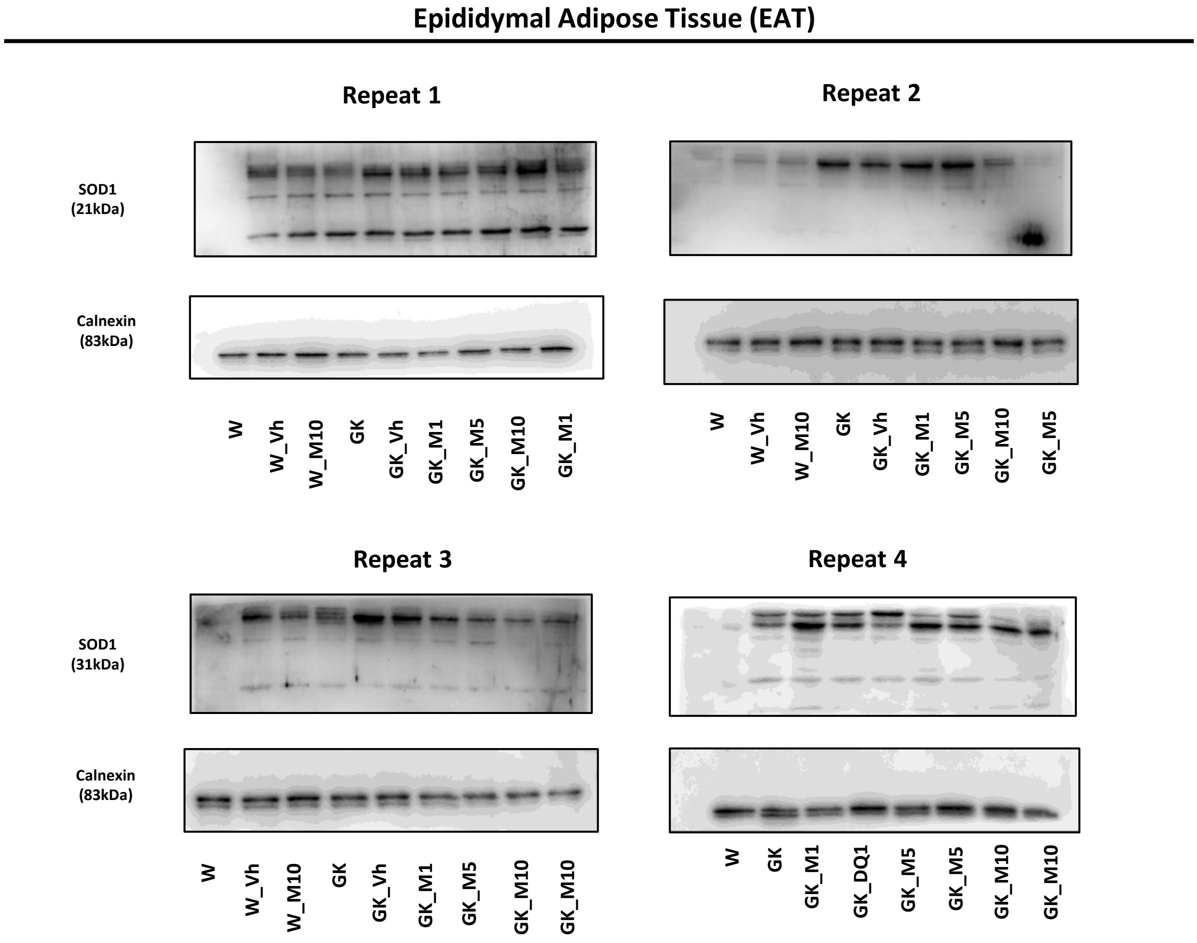

SOD1 [Figure 6B]

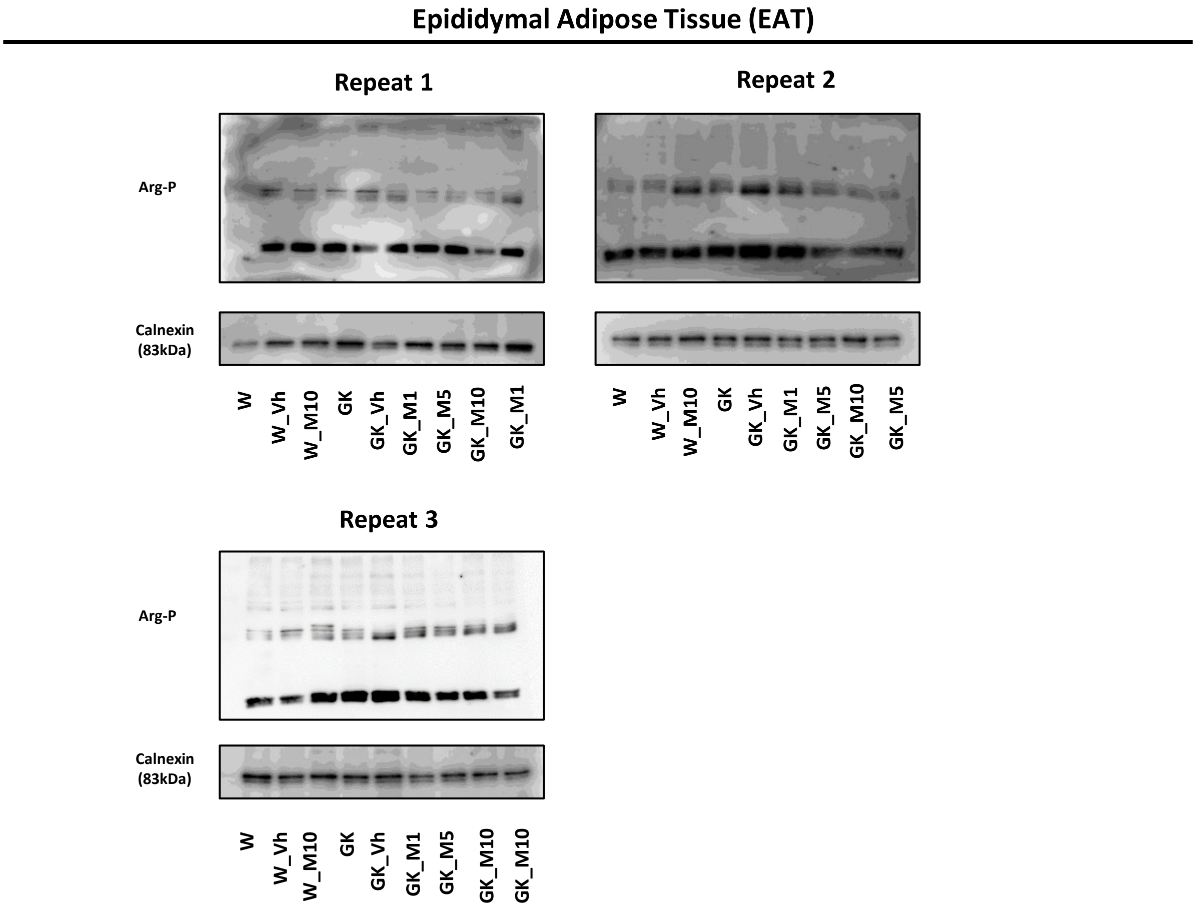

GLUT4 [Supplementary figure S4C]

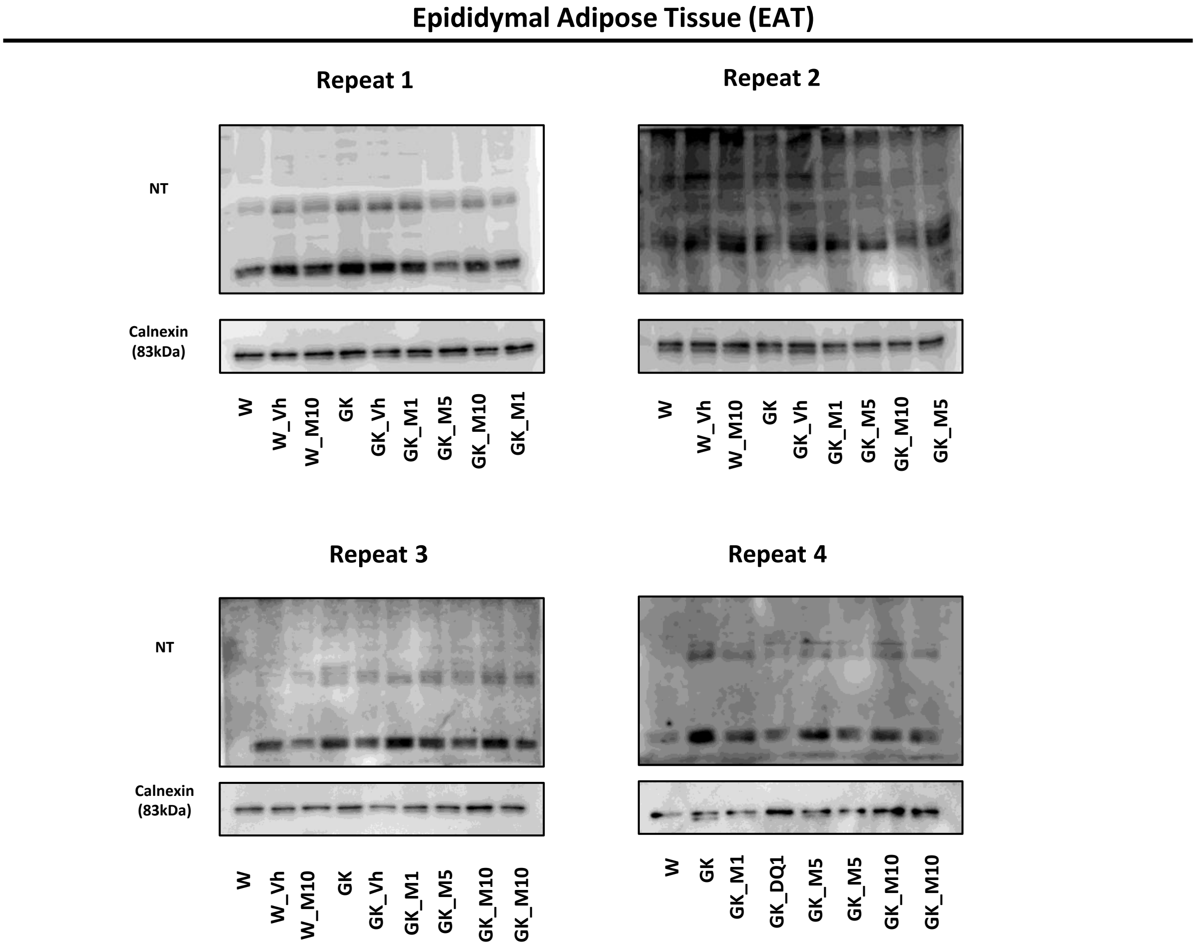

NT [Figure 6E]

Epididymal Adipose Tissue (EAT)

Repeat 1

PTP1B (50 kDa)

β-Actin (42 kDa)

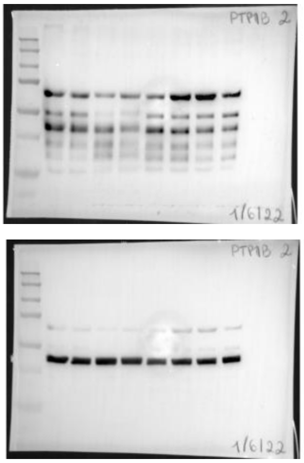

GK GK_Vh GK_M1 GK_M5 GK_M10 GK_M1 W W_Vh W_M10

Repeat 2

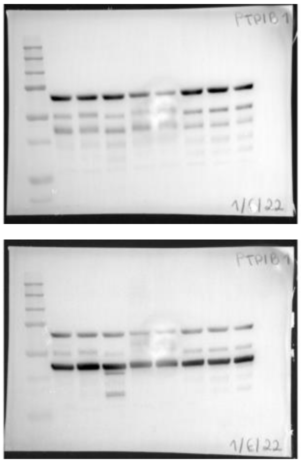

GK GK_Vh GK_M1 GK_M5 GK_M10 GK_M1 W W_Vh W_M10

Repeat 3

PTP1B (50 kDa)

β-Actin (42 kDa)

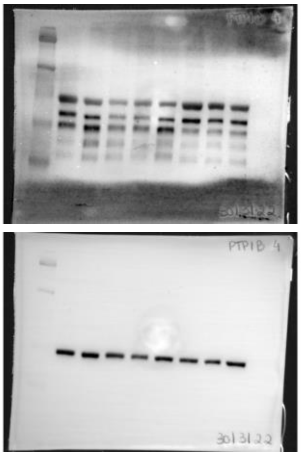

GK GK_Vh GK_M1 GK_M5 GK_M10 GK_M1 W W_Vh W_M10

Repeat 4

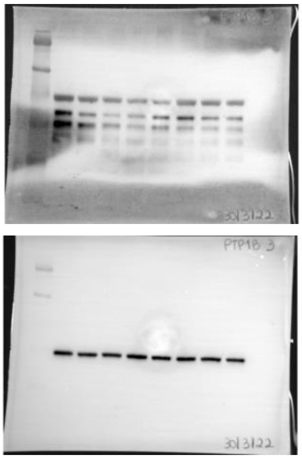

GK GK_Vh GK_M1 GK_M5 GK_M10 GK_M1 W W_Vh W_M10

PTP1B [Figure 5B]

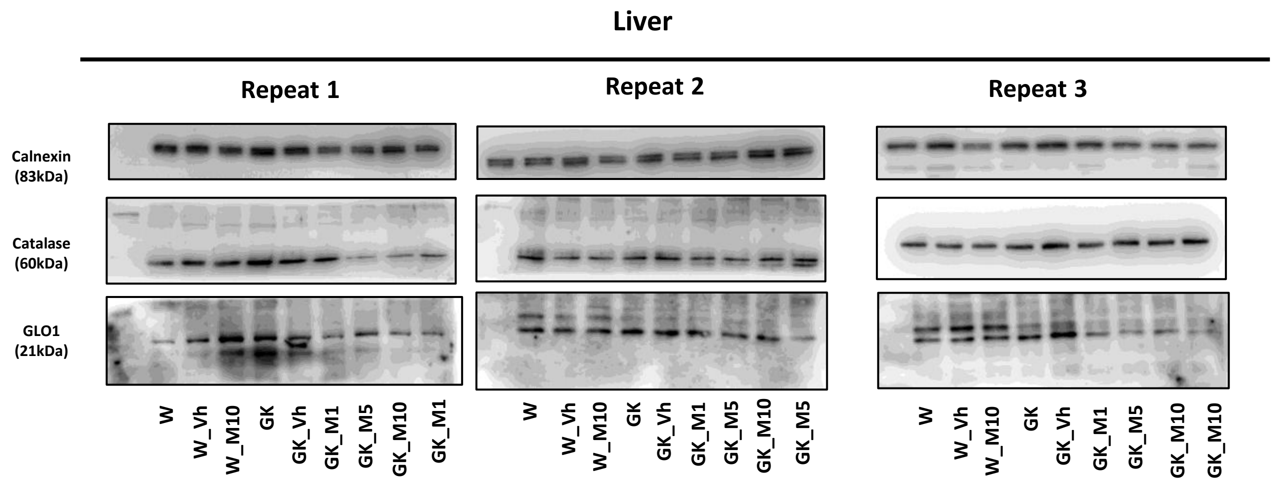

GLO1 [Figure 6F]

Catalase [Supplementary figure S5A]

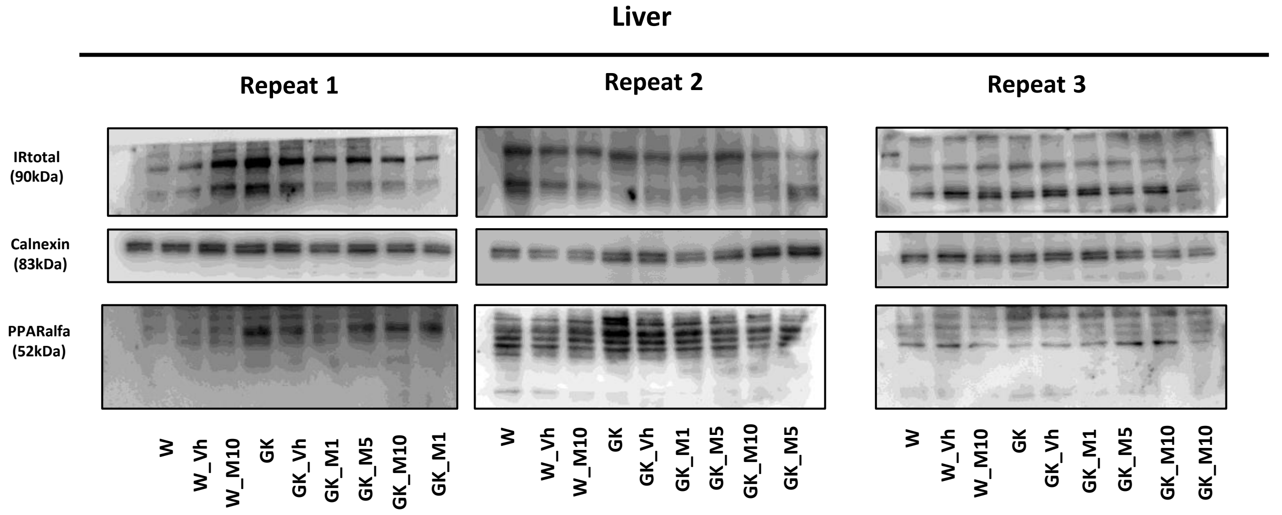

IRtotal [Supplementary figure S3A]
PPARalfa [Supplementary figure S3E]

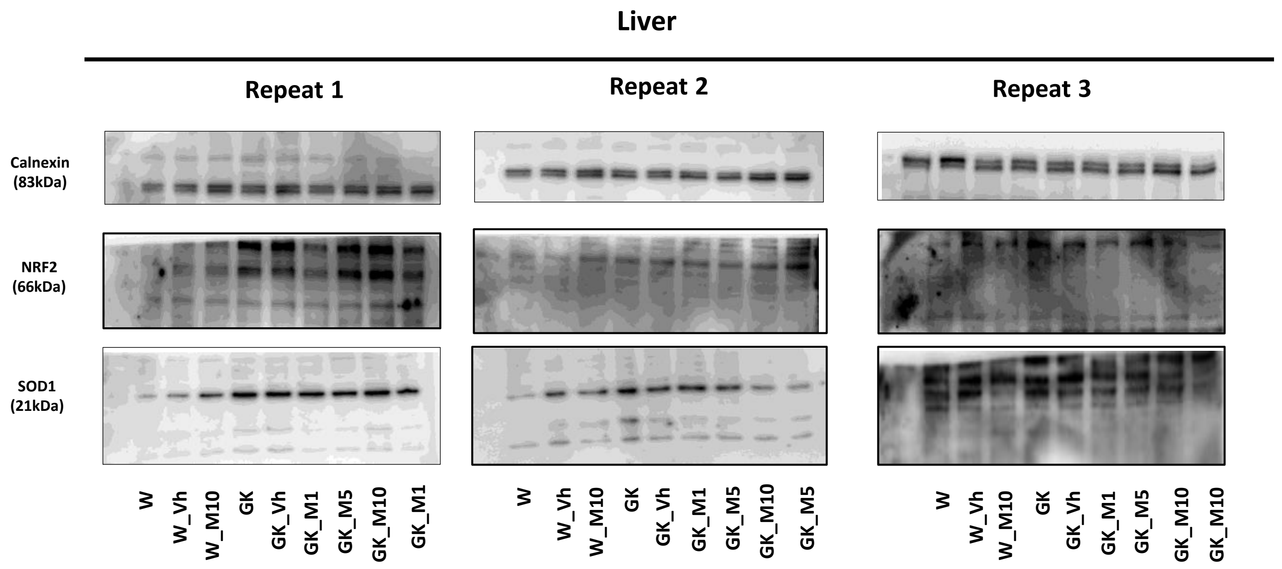

NRF2 and SOD1 [Supplementary figure S5B and S5C]

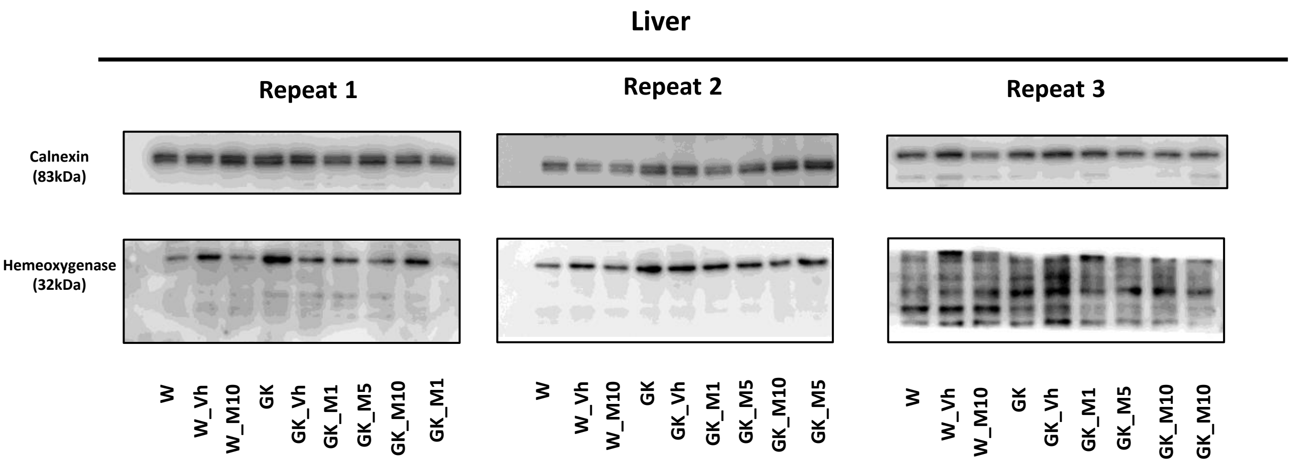

MG-H1 [Supplementary figure S5]

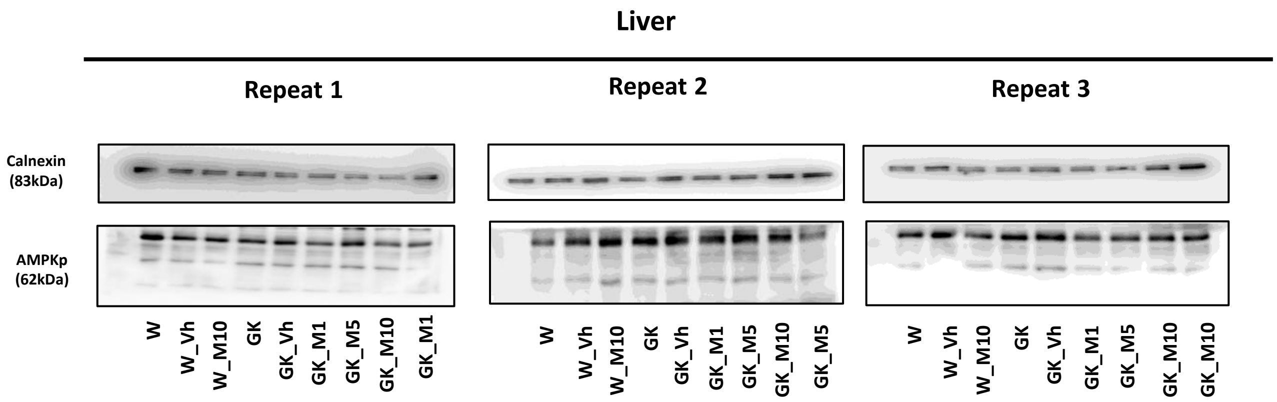

AMPKp [Supplementary figure S3C]

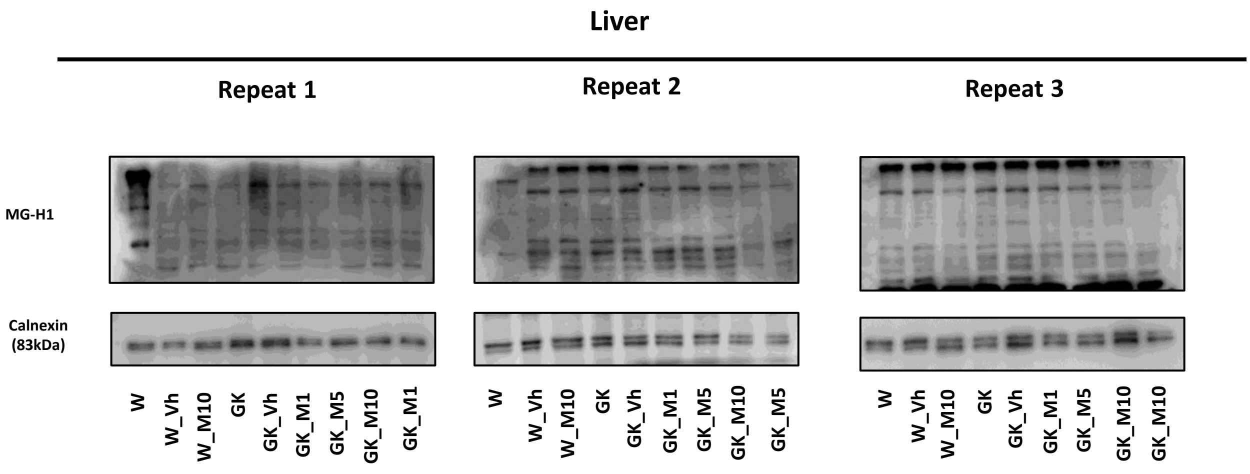

MG-H1 [Supplementary figure S5E]

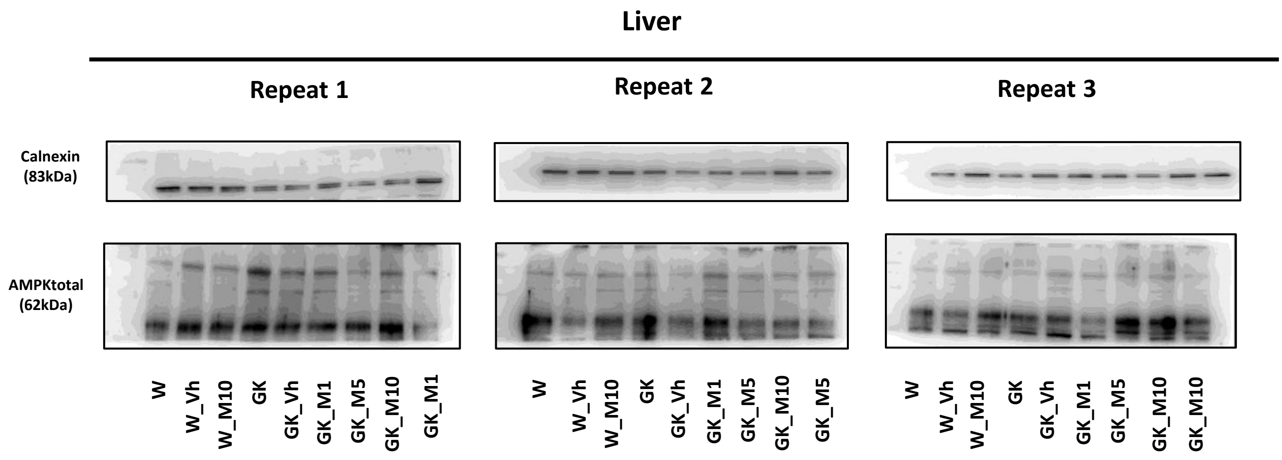

AMPKtotal [Supplementary figure S3B]

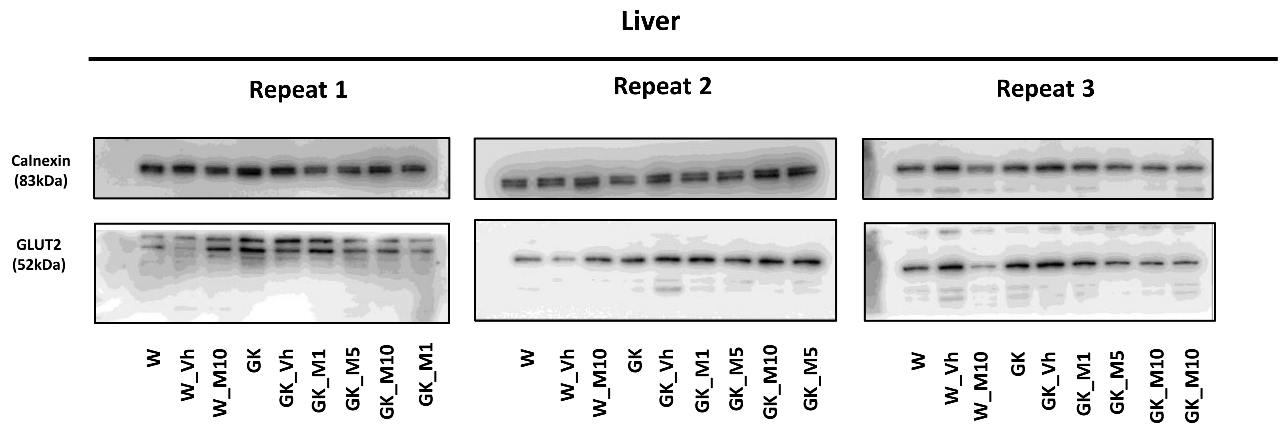

GLUT2 [Supplementary figure S3D]

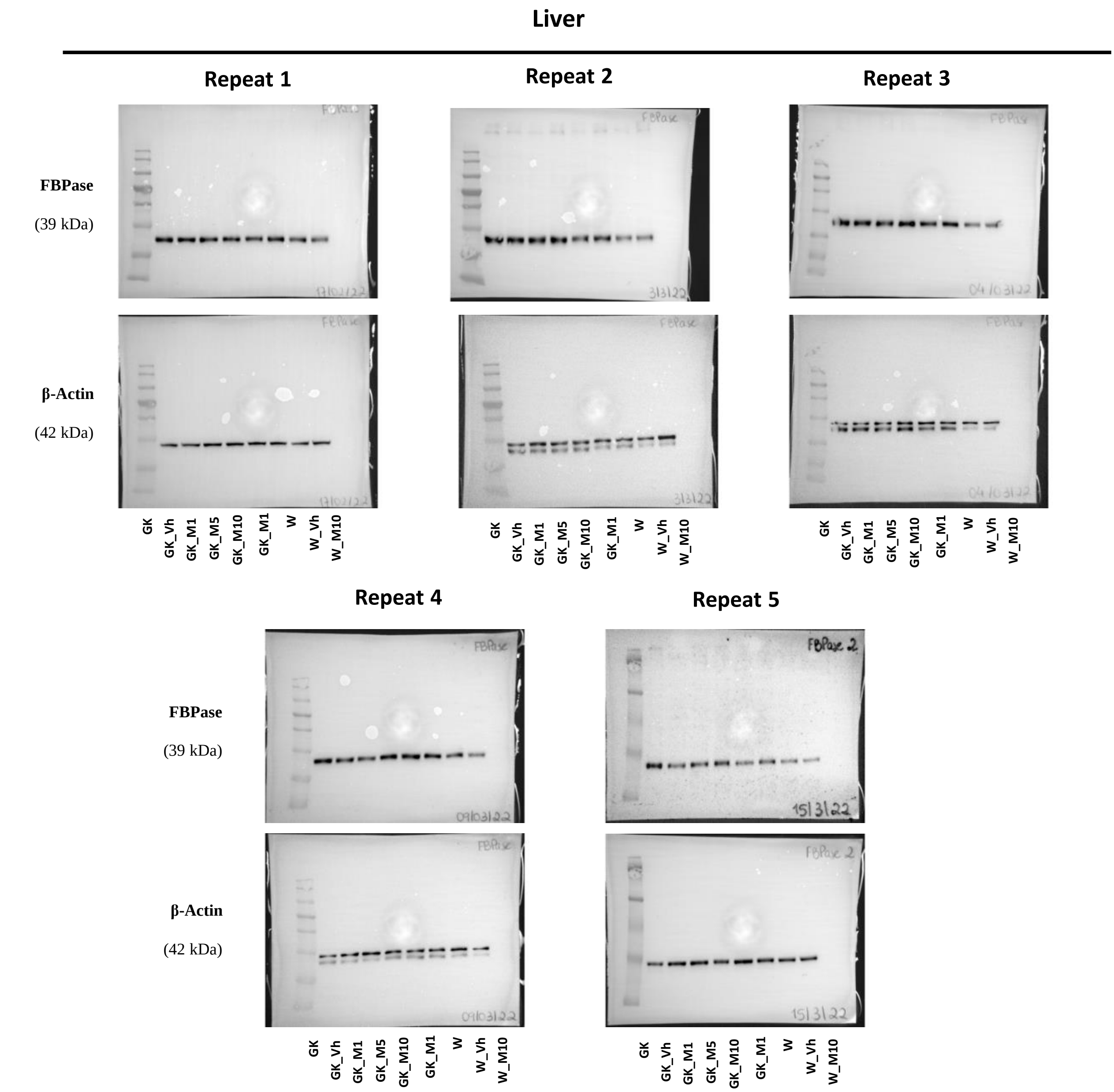

FBPase [Supplementary figure S3F]

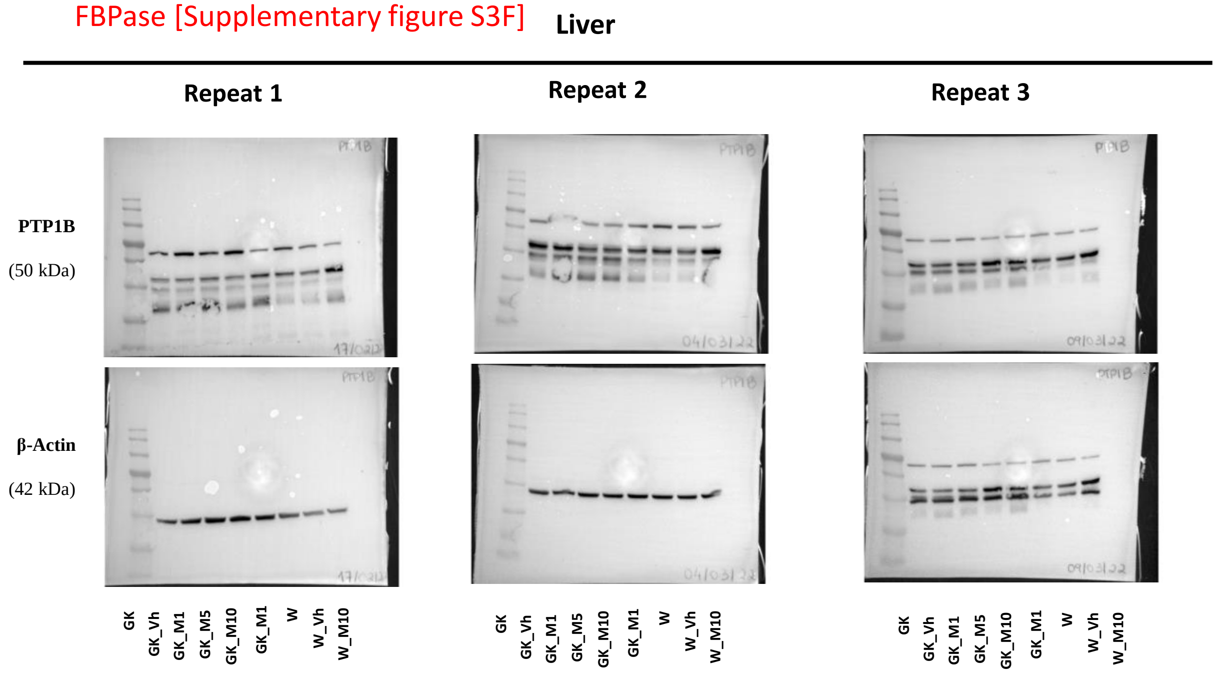

PTP1B [Figure 5C]

Heart

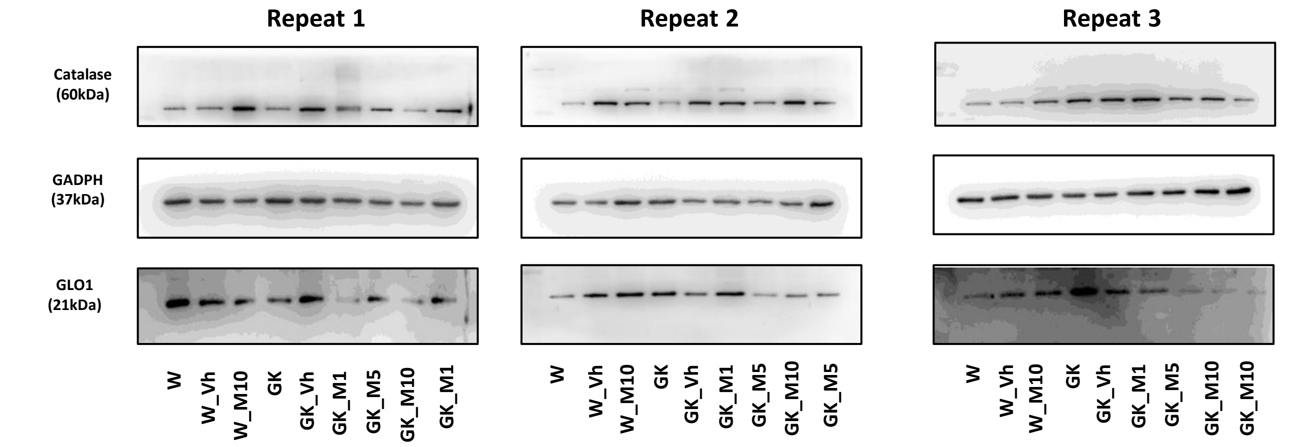

Catalase [Figure 7A]
GLO1 [Figure 7B]

Heart

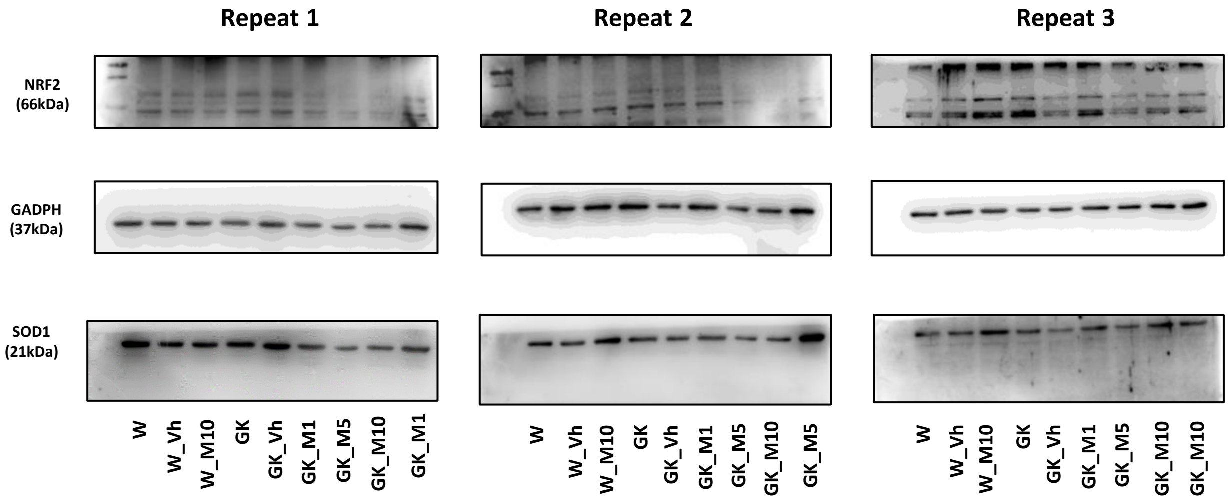

NRF2 and SOD1 [Supplementary figure S6A and S6B]

Heart

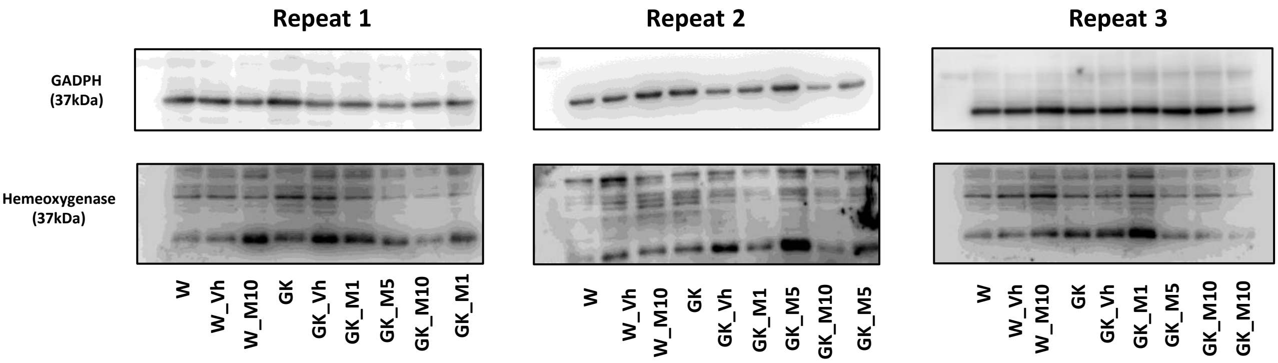

Hemeoxygenase [Supplementary figure S6C]

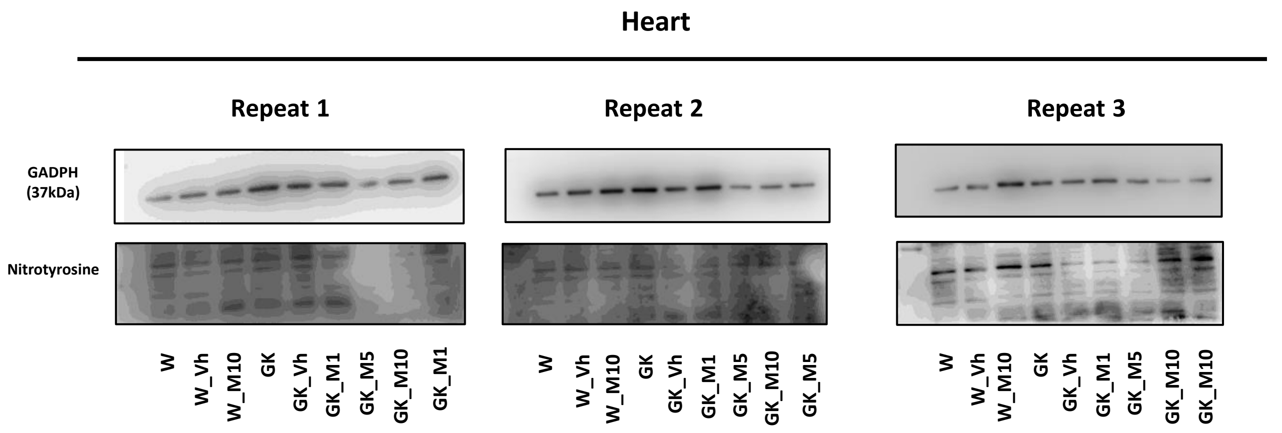

Nitrotyrosine[Supplementary figure S6D]

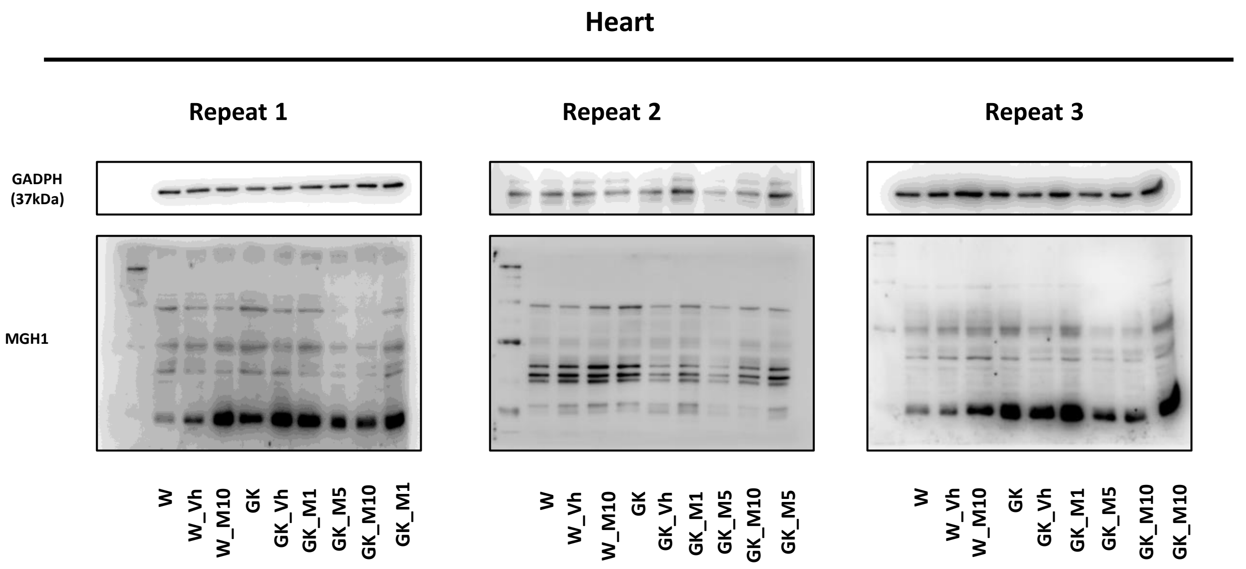

MG-H1 [Figure 7C]
