# Supplementary material for: Melanoxetin: A Hydroxylated Flavonoid Attenuates Oxidative Stress and Modulates Insulin Resistance and Glycation Pathways in an Animal Model of Type 2 Diabetes Mellitus
Source: Pharmaceutics. 2024 Feb 9;16(2):261. doi: 10.3390/pharmaceutics16020261 (PMC10892797; doi:10.3390/pharmaceutics16020261)
Supplement: Supplementary file 1 [file pharmaceutics-16-00261-s001.zip › pharmaceutics-2836090-supplementary.pdf]

Supporting Information

Melanoxetin: A Hydroxylated Flavonoid Attenuates Oxidative Stress and Modulates Insulin Resistance and Glycation Pathways in an Animal Model of Type 2 Diabetes Melli-tus

Sónia Rocha, Andreia Amaro, Marcos D. Ferreira-Junior, Carina Proença, Artur M.S. Silva, Vera M. Costa, Sara Oliveira, Diogo A. Fonseca, Sónia Silva, M. Luísa Corvo, Marisa Freitas, Paulo Matafome, Eduarda Fernandes

Table S1: Table detailing antibody names, dilutions, and respective purchase sources for Western Blot analyses.

Primary antibody	Secondary antibody	Dilution	Company
**Anti- β-Actin**	Mouse	1:1000	Merck
**Anti-AMPK total**	Rabbit	1:1000	Cell Signalling
**Anti-AMPKp**	Rabbit	1:1000	Cell Signalling
**Anti-Argpyrimidine**	Mouse	1:500	Nordic
**Anti-Calnexin**	Goat	1:1000	Sicgen
**Anti-Catalase**	Rabbit	1:1000	Abcam
**Anti-FBPase**	Rabbit	1:1000	Cell Signalling
**Anti-GADPH**	Goat	1:1000	Sicgen
**Anti-GLO1**	Rabbit	1:1000	Abcam
**Anti-GLUT2**	Mouse	1:1000	Abcam
**Anti-GLUT4**	Mouse	1:1000	Abcam
**Anti-Hemeoxygenase**	Mouse	1:1000	Abcam
**Anti-IR total**	Rabbit	1:1000	Santa Cruz
**Anti-MG-H1**	Mouse	1:500	HylcutBiotech
**Anti-Nitrotyrosine**	Mouse	1:500	Abcam
**Anti-Nrf2**	Mouse	1:1000	Santa Cruz
**Anti-PPARα**	Rabbit	1:1000	Abcam
**Anti-PPARγ**	Rabbit	1:1000	Cell Signalling
**Anti-PTP1B**	Rabbit	1:1000	Abcam
**Anti-SOD1**	Rabbit	1:1000	Abcam

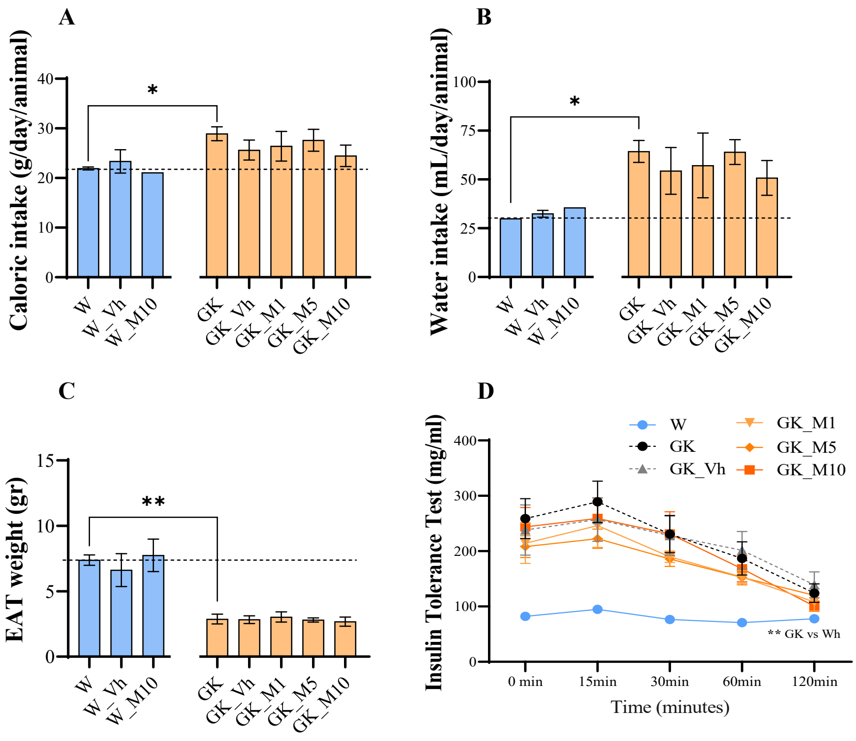

Figure S1: Effect of melanoxetin on caloric intake (A), water intake (B) EAT weight (C) and insulin tolerance test (D) in normal and diabetic animals. Results are expressed as mean ± SEM of 3 to 6 animals per group. **p*<0.05, ***p*<0.01.

Epididymal Adipose Tissue

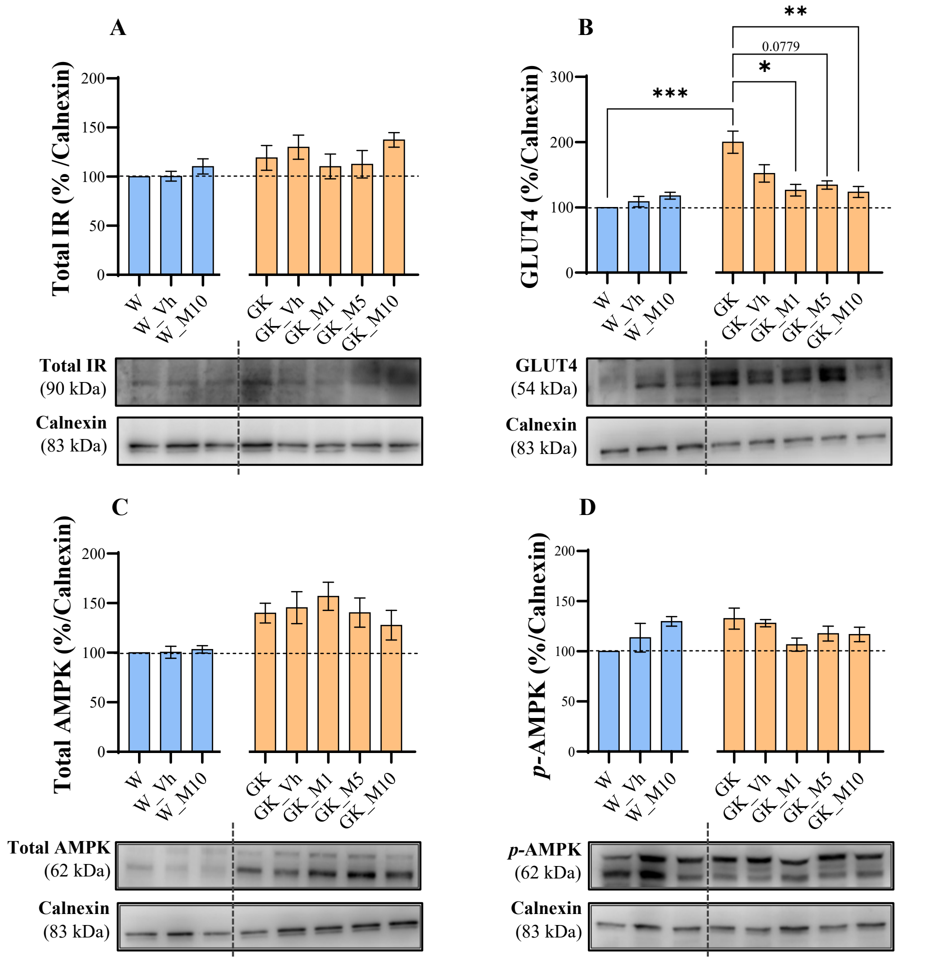

Figure S2: Effect of melanoxetin on the expression of total IR (A), GLUT4 (B), total AMPK (C), and *p*-AMPK (D) in epididymal adipose tissue. Results are expressed as mean ± SEM of 3 to 6 animals per group. *p<0.05, **p<0.01, ***p<0.001.

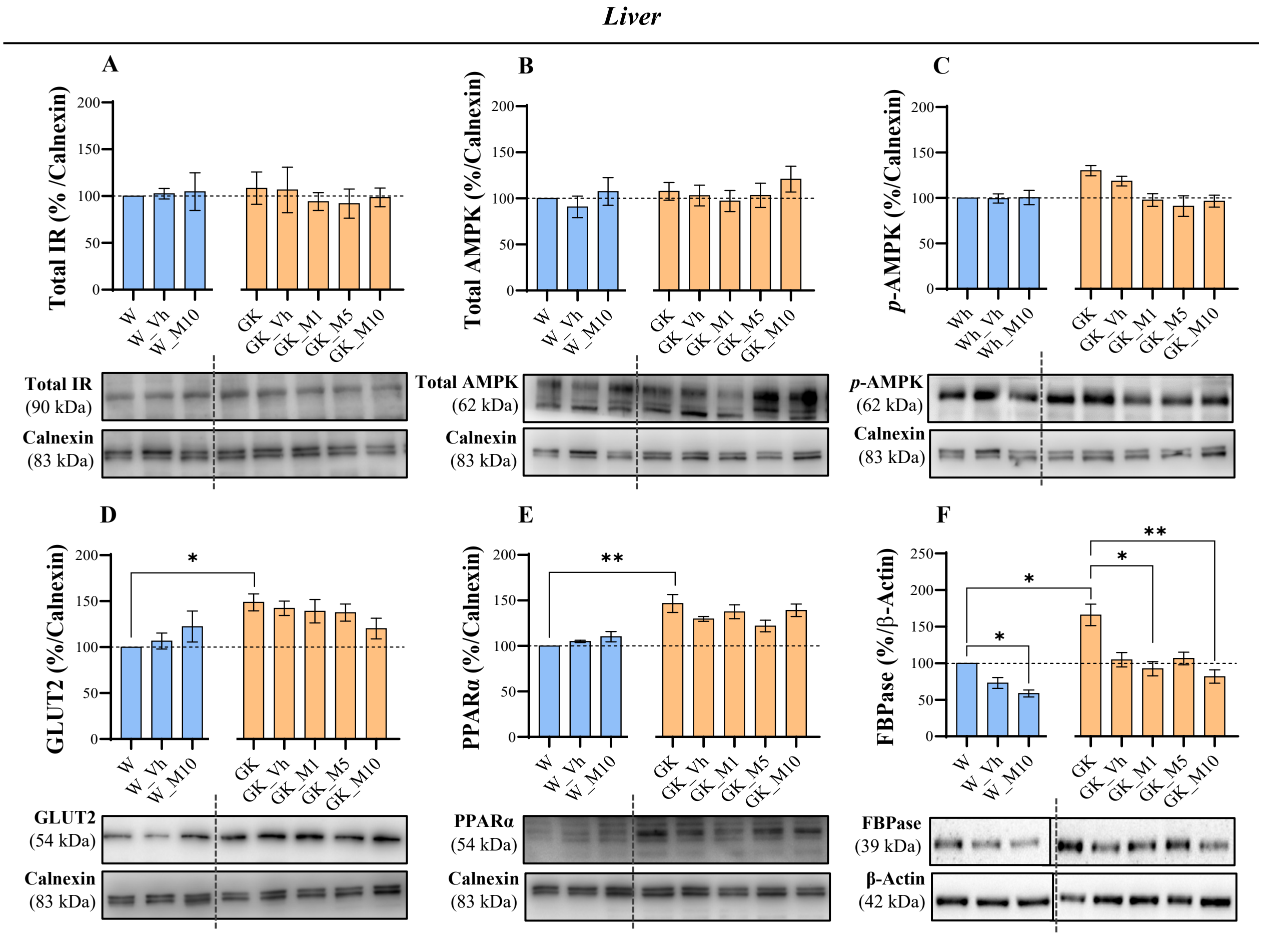

Figure S3: Effect of melanoxetin on the expression of Total IR (A), Total AMPK (B), *p*-AMPK (C), GLUT2 (D), PPARα (E) and FBPase (F) in liver tissue. Results are expressed as mean ± SEM of 3 to 6 animals per group. **p*<0.05, ***p*<0.01.

Epididymal Adipose Tissue

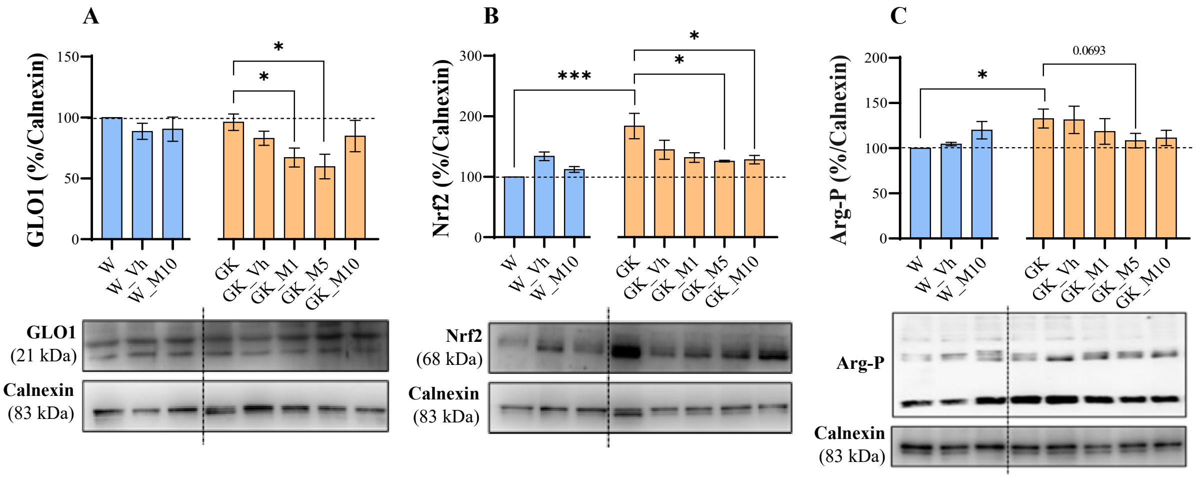

Figure S4: Effect of melanoxetin on the expression of GLO1 (A), Nrf2 (B) and Arg-P (C) in epididymal adipose tissue. Results are expressed as mean ± SEM of 3 to 6 animals per group. **p*<0.05, ****p*<0.001.

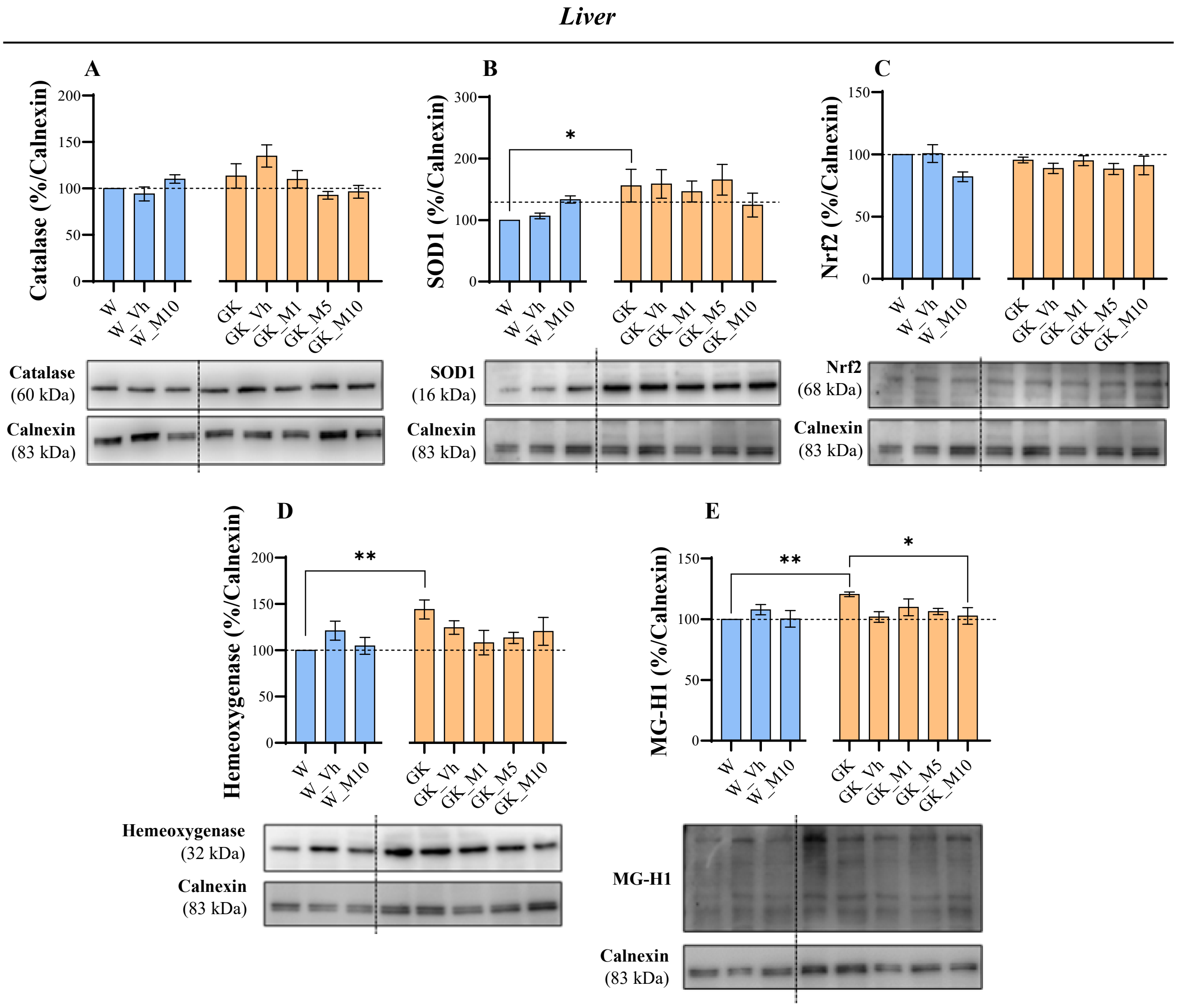

Figure S5: Effect of melanoxetin on the expression of catalase (A), SOD1 (B), Nrf2 (C), hemeoxygenase (D), and MG-H1 (E) in liver tissue. Results are expressed as mean ± SEM of 3 to 6 animals per group. **p*<0.05, ***p*<0.01.

Heart

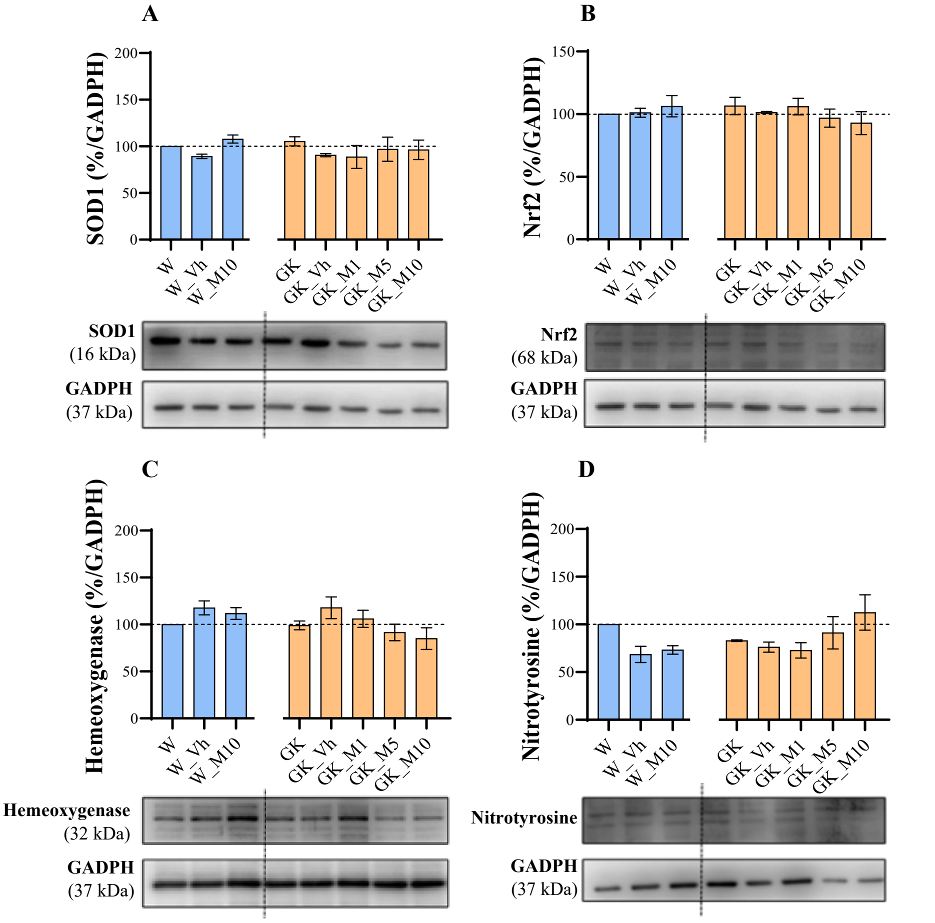

Figure S6: Effect of melanoxetin on the expression of SOD1 (A), Nrf2 (B), hemeoxygenase (C), and nitrotyrosine (D) in the heart. Results are expressed as mean ± SEM of 3 to 6 animals per group.

Aorta

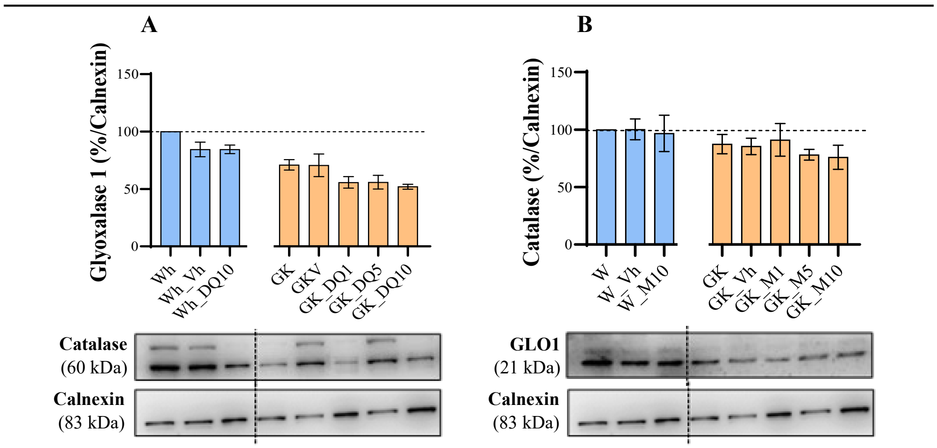

Figure S7: Effect of melanoxetin on the expression of GLO1 (A) and catalase (B) in the aorta. Results are expressed as mean ± SEM of 3 to 6 animals per group.

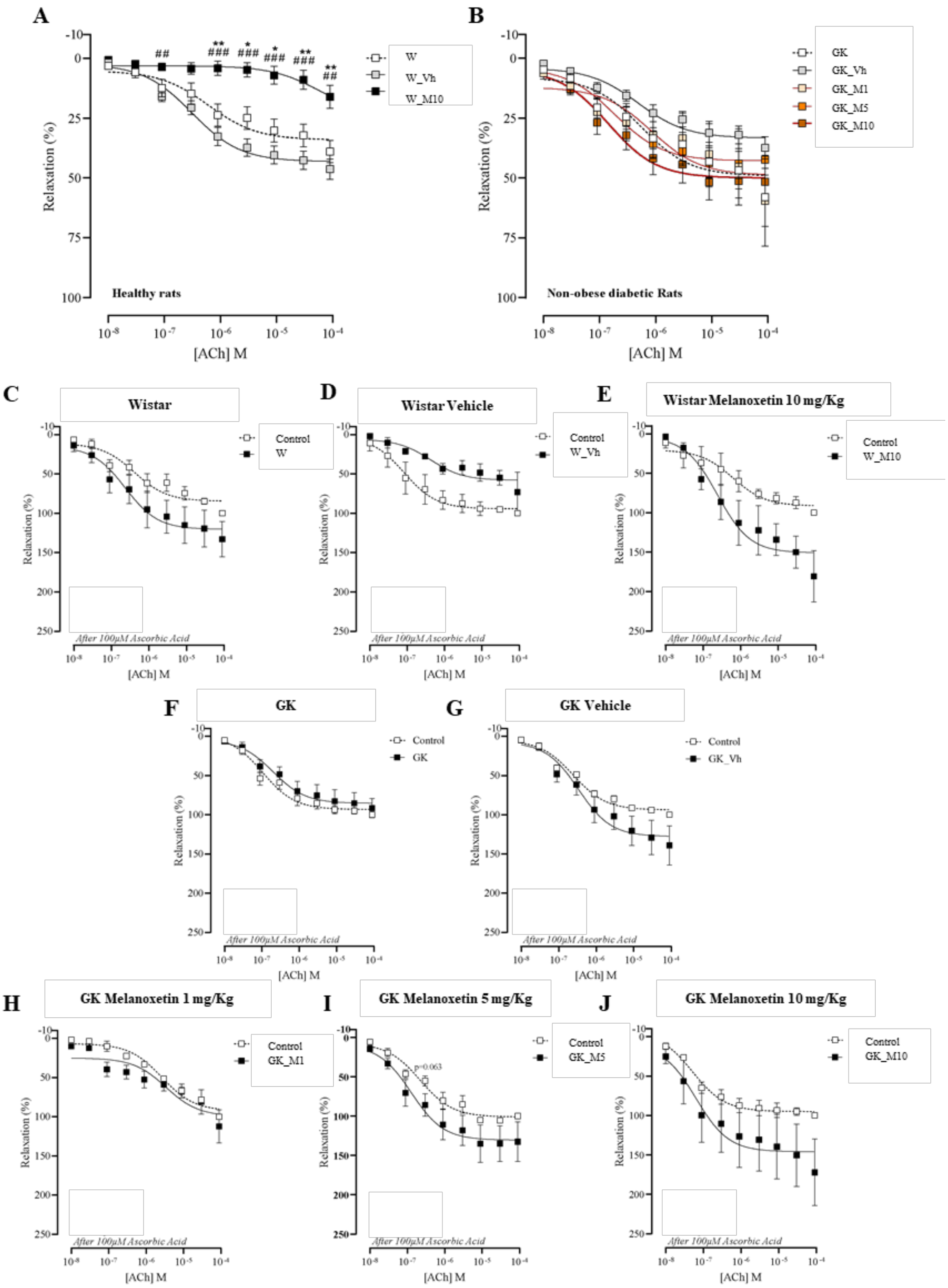

Figure S8: Effects of melanoxetin treatment on relaxation response of the isolated to acetylcholine (ACh). The relaxation response was measured before (A and B) and after pre-incubation with ascorbic acid from Wistar control and Wistar vehicle rats (C and D), Wistar rats treated with 10mg/kg of melanoxetin (E), GK control and vehicle (F and G) and GK rats administered with different concentrations of melanoxetin - 1mg/kg, 5mg/kg and 10mg/kg (H, I and J).
